# Metaproteomics reveals associations between microbiome and intestinal extracellular vesicle proteins in pediatric inflammatory bowel disease

**DOI:** 10.1038/s41467-018-05357-4

**Published:** 2018-07-20

**Authors:** Xu Zhang, Shelley A. Deeke, Zhibin Ning, Amanda E. Starr, James Butcher, Jennifer Li, Janice Mayne, Kai Cheng, Bo Liao, Leyuan Li, Ruth Singleton, David Mack, Alain Stintzi, Daniel Figeys

**Affiliations:** 1 Ottawa Institute of Systems Biology and Department of Biochemistry, Microbiology and Immunology, Faculty of Medicine, University of Ottawa, 451 Smyth Road, Ottawa, ON K1H 8M5 Canada; 2 Department of Paediatrics, Faculty of Medicine, University of Ottawa and Children’s Hospital of Eastern Ontario Inflammatory Bowel Disease Centre and Research Institute, 401 Smyth Road, Ottawa, ON K1H 8L1 Canada; 3 Department of Chemistry and Biomolecular Sciences, University of Ottawa, 10 Marie Curie, Ottawa, ON K1N 6N5 Canada; 4 Canadian Institute for Advanced Research, 661 University Ave, Toronto, ON M5G 1M1 Canada

## Abstract

Alterations in gut microbiota have been implicated in the pathogenesis of inflammatory bowel disease (IBD), however factors that mediate the host–microbiota interactions remain largely unknown. Here we collected mucosal-luminal interface samples from a pediatric IBD inception cohort and characterized both the human and microbiota proteins using metaproteomics. We show that microbial proteins related to oxidative stress responses are upregulated in IBD cases compared to controls. In particular, we demonstrate that the expression of human proteins related to oxidative antimicrobial activities is increased in IBD cases and correlates with the alteration of microbial functions. Additionally, we reveal that many of these human proteins are present and show altered abundance in isolated free extracellular vesicles (EVs). Therefore, our study suggests that the alteration of intestinal EV proteomes is associated with the aberrant host–microbiota interactions in IBD.

## Abstract (web summary)

Gut microbial dysbiosis has been implicated in the pathogenesis of inflammatory bowel disease. Here, the authors examine host-microbiota protein interactions that occur in inflammatory bowel disease; they show an upregulation in proteins related to antimicrobial activities, and alterations in intestinal extracellular vesicles that are associated with aberrant microbiota-interactions.

## Introduction

Inflammatory bowel disease (IBD) is a condition characterized by inflammation of the mucosal lining of the intestinal tract. Crohn’s disease (CD) and ulcerative colitis (UC) are the two principal subtypes of IBD. Both subtypes can display similar clinical manifestations and follow similar treatment strategies at times, but they also can vary due to fundamental differences in the diseases^1^. It has been estimated that >0.3% of the population in many developed countries suffer from IBD, with the incidence in developing countries on the rise^2^. In particular, the worldwide incidence of pediatric-onset IBD is increasing^3^. Childhood-onset IBD is distinct from adult-onset IBD with differences observed in progression, anatomical location of disease, and treatment outcomes^4^. Childhood-onset IBD often leads to growth failure and has serious long-term health consequences that result in higher health-care costs^5^. Hence, there is an urgent need to fully understand the pathogenesis of childhood-onset IBD to allow for precision therapeutics and long-term disease management.

Although the exact mechanism underlying the development of IBD remains unclear, its pathogenesis is linked to multiple factors including genetic susceptibility, immune activity, environmental factors (such as diet and life style), and the intestinal microbiome^4^. Accumulating evidence has revealed alterations of the gut microbiota in both adults and children with IBD^6–8^. However, most studies that characterize the microbiota in IBD patients use stool as proxy for sampling the gut microbiota. It has become increasingly clear that the mucosal-associated microbial dysbiosis in pediatric CD patients is only weakly reflected in fecal samples^6^ and unfortunately limited studies have been able to sample at the site of disease within the gastrointestinal tract^9,10^. We previously demonstrated an important role of mitochondrial dysfunction and an aberrant host-microbiota crosstalk in the pathogenesis of CD using mucosal-luminal interface (MLI) aspirates^9^. This study highlighted the value of sampling directly at the site of disease in order to elucidate the complex microbiota-host interactions and their implications in IBD. In addition, the study of a pediatric IBD inception cohort also mitigates cofounding factors such as medication, lifestyle and disease complications which are associated with the study of adult patients with long-standing IBD.

Deep profiling of the microbiota composition and functions is typically achieved through DNA and RNA sequencing^11^, However, since gene/transcript presence does not necessarily indicate protein expression, metaproteomics, which directly measures the expressed proteins, can provide more precise functional information^12^. Moreover, mass spectrometry (MS)-based proteome analysis allows simultaneous measurement of proteins of both human and microbiota origin^13^, and hence is a promising approach to aide in deciphering host-microbe interactions in complex intestinal eco-systems. Limited metaproteomic studies of stool and MLI samples from adult IBD patients have been previously reported^10,14^ and there are no metaproteomic studies focusing on pediatric IBD patients. Therefore, in this study, we took advantage of the MLI aspirate sampling approach, high-resolution MS and our previously established MetaPro-IQ bioinformatic workflow^15^ to study the proteomes of both microbiota, human and the isolated extracellular vesicles (EVs) present at the MLI of children with new-onset IBD. We characterized the functional activity of microbiome and identified microbial functions and metabolic pathways that were upregulated in IBD compared to control and not previously reported, including those in response to oxidative stress, such as DNA damage repair/defense, mobilome, and l-cysteine degradation. We also showed that the intestinal commensal bacterium *Faecalibacterium prausnitzii* was associated with the CD at strain level. Importantly, we further demonstrated for the first time that the host defense proteins, including those for producing reactive oxidants, were present in the intestinal EVs and correlated with the functional alterations of microbiota in pediatric IBD.

## Results

### Pediatric IBD cohort and metaproteomic characterization

In this study, we enrolled 71 treatment-naïve pediatric patients, including 25 CD, 22 UC, and 24 non-IBD control subjects that showed no macroscopic-intestinal inflammation (Table 1; details in Supplementary Data 1). No significant difference in age was observed between control, CD or UC (*p* = 0.7588; Kruskal–Wallis test), nor did we observed a significant difference in the number of females and males in each group (control, *p* = 0.5413; CD, *p* = 0.2295 and UC, *p* > 0.9999; Binomial two-tailed test). The majority (81.2%) of pediatric UC participants had extensive disease, defined as a Paris classification of E3 or E4, which is consistent with previous studies^16^. Furthermore the majority (80%) of CD patients presented with colonic or ileocolonic disease, also in accordance with previous reports^17^.

**Table 1:** Patient characteristics of treatment-naïve pediatric IBD cohort

	Controls (*n* = 24)	CD (*n* = 25)	UC (*n* = 22)
Gender			
Male/female	10/14	16/9	11/11
Age			
Median (Q1–Q3)	15.0 (13.5–16.3)	13.8 (12.3–16.1)	14.9 (11.2–16.2)
Disease activity			
Severe/moderate/mild	NA	(13/6/6)	(5/14/3)
Paris classification			
A1a/A1b/A2	NA	(4/19/2)	NA
L1/L2/L3/L4a/L4b	NA	(3/4/16/12/4)	NA
B1/B2/B3/p	NA	(23/1/1/4)	NA
G0/G1	NA	(17/8)	NA
E1/E2/E3/E4	NA	NA	(1/3/4/14)
S0/S1	NA	NA	(14/8)
MLI aspirate samples (inflammatory status)			
DeC (w/o inflam/w inflam)	20/0	11/9	17/1
AsC (w/o inflam/w inflam)	22/0	11/11	12/6
TI (w/o inflam/w inflam)	21/0	16/3	16/0

**Fig. 1:**
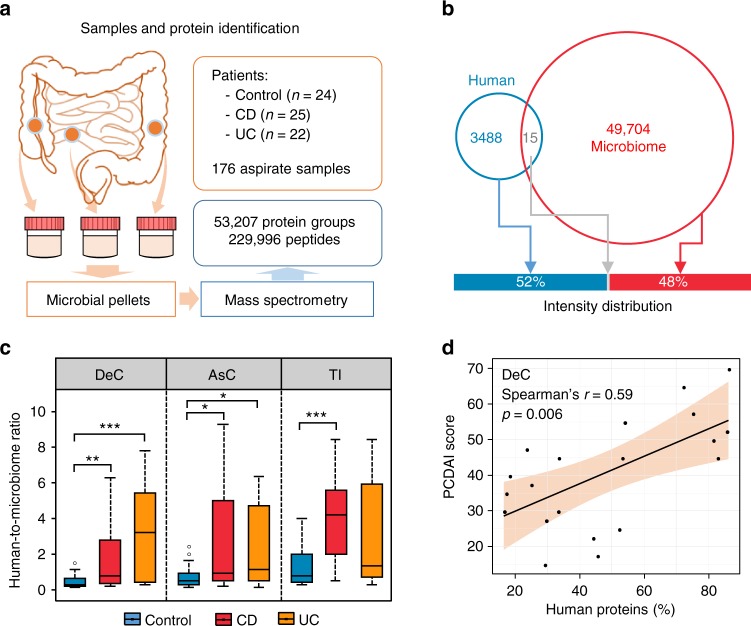
Metaproteomics approach for assessing both human and microbiota proteins in intestinal aspirate samples. **a** Experimental workflow; **b** Distribution of quantified human- and microbiome-derived protein groups. Venn diagram indicates the numbers of quantified protein groups, while the bar graph shows the intensity of human or microbiome proteins. **c** Human-to-microbiome ratios of total protein intensity. For the box plot, the bottom and top of the box are the first and third quartiles, respectively. The middle line represents the sample median. Whiskers are drawn from the ends of the interquartile range (IQR) to the furthest observations within 1.5 times the IQR range. Outliers >1.5 times the IQR are indicated with circle. *p < 0.05, **p < 0.01, ***p < 0.001. **d** Representative scatter plot between the percentage of human proteins and CD disease severity score

We used metaproteomics to characterize the functionality of intestinal microbiome from 176 MLI aspirate samples collected from the descending colon (DeC), ascending colon (AsC), or terminal ileum (TI), either with or without inflammation, at the time of diagnostic colonoscopy (Table 1 and Fig. 1a). We quantified 229,996 unique peptides and 53,207 protein groups in these 176 samples, with an average identification rate of 39 ± 8% (mean ± SD). On average, 32,882 ± 8836 unique peptides and 14,603 ± 3328 protein groups were identified per sample. There were 226 that showed biogeographic site-specific expressions (Supplementary Note 1, Supplementary Fig. 1 and Supplementary Data 2). To ensure consistent MS performance, a quality control (QC) sample was generated by combining 10 randomly selected MLI samples; this sample was then repeatedly run in every MS batch analysis. Sample-wise Pearson correlations of the quantified proteins showed that there was no obvious sample clustering based on MS date, and the eight QC samples closely clustered together (with Pearson’s *r* of 0.86 ± 0.02; Supplementary Fig. 2). These results indicate reproducible MS measurements and metaproteomic workflow in this study.

### Intestinal MLI metaproteome landscape alterations in IBD

One advantage of metaproteomics is that both microbiota and host proteins can be measured in a single analysis. Among the 53,207 protein groups quantified, 93% were from intestinal microorganisms, with only 3488 (7%) protein groups derived from the host. However, human proteins constituted 52% of the total protein intensities measured in the MLI samples (Fig. 1b), indicating that the MLI microbiome was associated with abundant host proteins. Our sample preparation workflow is also similar to procedures for isolating shed microvesicles (100–1000 nm), which often employ a 10,000–20,000 g centrifugation step^18^, and this could partially explain the high levels of human proteins in our samples. In evaluating the ratios between human and microbial proteins in MLI aspirate samples, we found that the samples from both CD and UC patients had an elevated proportion of human proteins (Fig. 1c). Furthermore, the percentage of human proteins significantly correlated with the clinical disease severity in CD (*p* = 0.006, 0.026, and 0.125 for DeC, AsC, and TI, respectively; Fig. 1d) but not in UC (*p* = 0.152, 0.167, and 0.052 for DeC, AsC, and TI, respectively).

**Fig. 2:**
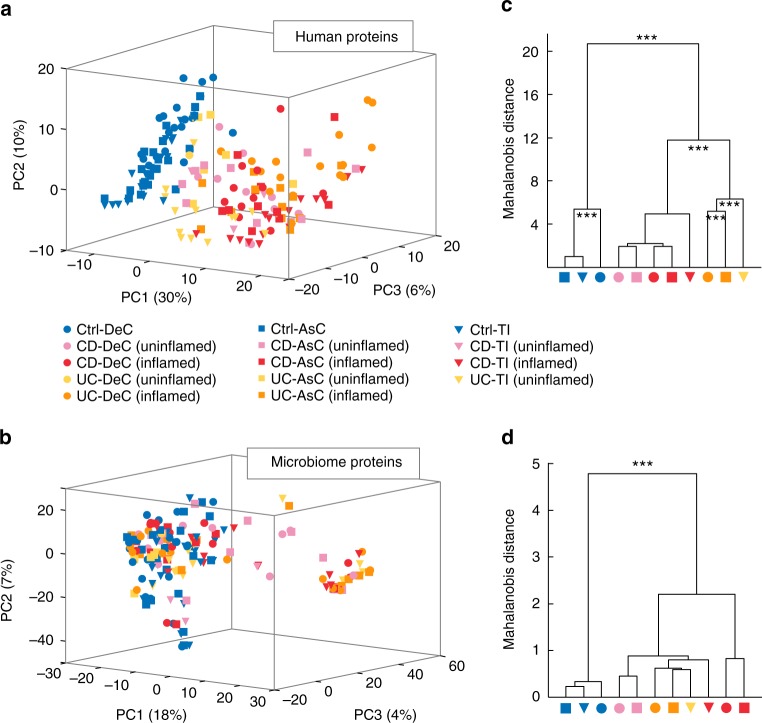
MLI host and microbiota proteome landscape alterations in pediatric IBD. **a** PCA score plot of MLI human-derived proteins quantified in ≥50% of the samples; **b** PCA score plot of MLI microbiome-derived proteins quantified in ≥ 50% of the samples; **c**, **d** Group mean clustering based on Mahalanobis distances calculated with MANOVA test using the first 10 PCs of PCA on MLI human-derives proteins (**c**) and MLI microbiome-derives proteins (**d**). The clusters are computed by applying the single linkage method to the matrix of Mahalanobis distances between group means. ***p < 0.001

Principal component analysis (PCA) was performed using the MLI human-derived proteins or non-human proteins (i.e. microbiota-derived) quantified in ≥50% of the samples. The PCA score plot using the MLI human proteins showed an obvious separation between control and IBD along the first component (PC1), while no obvious difference was observed between CD and UC (Fig. 2a). In addition, the IBD samples emanating from intestinal areas without macroscopic inflammation tend to locate between those of controls and inflamed areas (Fig. 2a and Supplementary Fig. 3). By contrast, the PCA analysis of microbiota-derived proteins showed that the control subjects were clustered together with most of the IBD subjects, and only a subset of IBD samples were shifted from the control. No obvious segregation corresponding to intestinal locations, or the presence of inflammation, was observed (Fig. 2b and Supplementary Fig. 3). Within-group metaproteome variations were evaluated using the pairwise Bray-Curtis dissimilarity index, which demonstrated lower within-group heterogeneity in controls as compared to both CD and UC (Supplementary Fig. 4). Power calculations using pairwise-distance matrix and PERMAOVA^19^ suggested that we were sufficiently powered (>99%) to detect relevant between-group differences (effect sizes/P-values were 0.21/0.001, 0.19/0.001, 0.27/0.001 for human-derived proteins, and 0.09/0.001, 0.07/0.001, 0.10/0.001 for microbial proteins, at DeC, AsC, and TI, respectively).

To further study whether significant differences corresponded to disease, presence of inflammation, or intestinal location, we performed multivariate analysis of variance (MANOVA) to compare the multivariate means of subgroups (CD-TI-uninflamed, UC-DeC-uninflamed and UC-AsC-uninflamed were not included due to insufficient sample size). MANOVA analysis of human-derived proteins yielded the greatest differences, which revealed significant differences (*p* < 0.001) for the comparisons of Control vs. IBD, CD, vs. UC, as well as the different intestinal locations in UC and Control groups (Fig. 2c). By contrast, MANOVA analysis of microbiota-derived proteins only yielded a significant difference between IBD and control, and no significant difference was observed between samples emanating from inflamed and uninflamed areas within IBD (Fig. 2d). Therefore, to identify IBD-related host and microbiota alterations, we combined samples from inflamed and uninflamed areas in IBD for further analysis.

### Elevated microbial response to oxidative stress in IBD

**Fig. 3:**
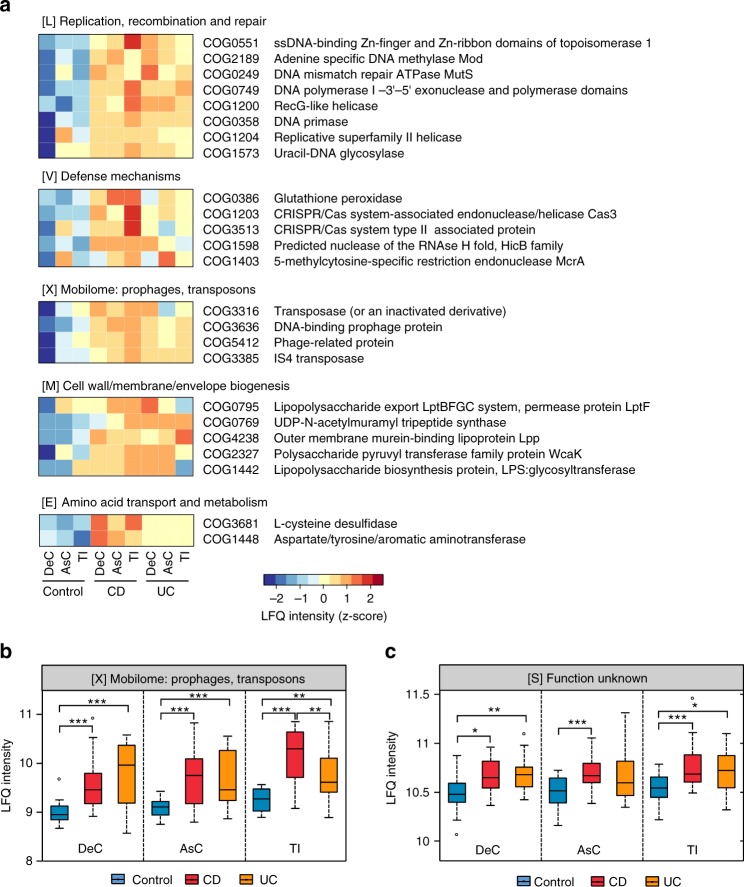
Functional compositions of MLI microbiome in pediatric IBD patients. **a** Heatmap of differentially abundant COGs in IBD. Representative COG categories are shown and the colors indicate the average LFQ intensity for each subgroup of samples. Each row corresponds to a COG with the COG id and name indicated. **b**, **c** LFQ intensity of COG category X (mobilome) and category S (unknown function) in MLI microbiome of IBD patients. For the box plot, the bottom and top of the box are the first and third quartiles, respectively. The middle line represents the sample median. Whiskers are drawn from the ends of the IQR to the furthest observations within the 1.5 times the IQR range. Outliers >1.5 times the IQR are indicated with circle. *p < 0.05, **p < 0.01, ***p < 0.001

In order to assess the microbial functions, we annotated all the quantified microbial proteins using the COG database, obtaining 2251 COGs from 24 COG categories. PLS-DA was performed to identify differentially abundant functions in IBD, analyzing the three intestinal locations individually (the goodness of prediction of PLS-DA model, *Q*^2^ = 0.58 for DeC, *Q*^2^ = 0.48 for AsC, *Q*^2^ = 0.55 for TI), which identified 71, 61, and 63 differentially abundant COGs for the DeC, AsC, and TI, respectively (Supplementary Data 3). There were 54 COGs identified with a VIP ≥ 2 in at least two intestinal locations and 42 COGs that were identified in all three locations; all were elevated in IBD compared to control. Functions related to the DNA replication, recombination, and repair (8 COGs in category L), bacterial outer membrane component biogenesis (5 COGs in category M), defense mechanisms (5 COGs in category V) and microbial mobilome (4 COGs in category X) were among the most significantly increased functions identified in IBD (Fig. 3a). We found that DNA damage repair proteins including those involved in base excise repair (BER) and mismatch repair (MMR) pathways, such as uracil-DNA glycosylase (UDG) and DNA MMR ATPase MutS, were increased in microbiome of pediatric IBD patients. In addition, we also revealed the increase of glutathione peroxidase, a microbial defense response protein induced under oxidative stress conditions^20^, in IBD compared to control (Fig. 3a).

At the COG category level, we found that mobilome and category S (function unknown) were significantly increased in IBD compared to controls (Fig. 3b, c), in particular the mobilome displayed significant increase in both CD and UC at all three intestinal regions. Four COGs belonging to mobilome were identified as differentially abundant in IBD microbiome compared to that of controls (Fig. 3a), among which 2 were transposase and the other 2 were phage-related proteins. Furthermore, we found that 2 COGs with functions relating to CRISPR/Cas system, an important prokaryotic immune system against foreign nucleic acids, such as phage genome sequences^21,22^, were significantly increased in the microbiome of pediatric IBD patients (Fig. 3a).

### Altered microbial sulfur and cysteine metabolism in IBD

**Fig. 4:**
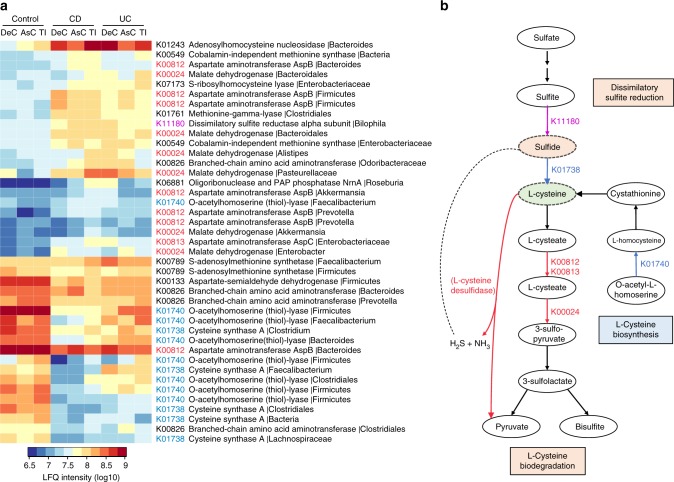
Alterations of sulfur and cysteine/methionine metabolism pathways in the MLI microbiome of pediatric IBD patients. **a** Taxon-specific sulfur or cysteine/methionine metabolism-related functions that were significantly changed in IBD. Heatmap shows the average LFQ intensity for each subgroup of samples. Each row represents one protein group, and the corresponding KO id, KO name and taxonomic assignment were indicated in the right panel. **b** Pathways relating to H_2_S production, l-cysteine biosynthesis, and degradation as represented by the identified differentially abundant proteins. Pathway was adapted from KEGG pathways (map00270 and map00920, version 5/8/2017)

Sulfate reducing bacteria and the production of hydrogen sulfide (H_2_S) in the gut are known to be increased in IBD patients^9,23^, however, the pathways underlying the production of H_2_S in the microbiome of pediatric IBD patients remain largely unknown. Therefore, we investigated proteins involved in H_2_S production, including those for l-cysteine metabolism enzymes as l-cysteine can act a substrate for H_2_S production. We found that l-cysteine desulfidase, an enzyme that catalyzes the production of H_2_S and ammonia from l-cysteine^24^, was significantly increased in IBD at both the DeC and AsC (Fig. 3a). KEGG annotation identified 60 KOs corresponding to 912 proteins that belong to sulfur metabolism or cysteine/methionine metabolism pathways. The wilcoxon rank-sum test identified 41 proteins that were significantly different between either CD or UC and controls in at least two intestinal locations, among which there were 18 proteins that decreased and 23 that increased in IBD (Fig. 4a). Within these 41 proteins, 13 are directly involved in the synthesis of l-cysteine, including 8 O-acetylhomoserine (thiol)-lyase proteins (K01740) and 5 cysteine synthase A proteins (K01738) (Fig. 4b). We found that 7 out of the 8 O-acetylhomoserine (thiol)-lyase proteins (including one from *Faecalibacterium*) and all 5 cysteine synthase A proteins were decreased in IBD. We also identified 14 microbial proteins involved in the degradation pathways of l-cysteine to generate hydrogen sulfite and pyruvate, including 8 aspartate aminotransferase (AST) proteins (K00812 or K00813) and 6 malate dehydrogenase (MDH) proteins (K00024) (Fig. 4b). All 6 MDH proteins and 7 of 8 AST proteins were increased in IBD. In addition, we found that a major component of dissimilatory sulfate reduction pathway, namely dissimilatory sulfite reductase alpha subunit (DsrA) from *Bilophila*, was significantly increased in the AsC/TI of CD and the DeC of UC patients (Fig. 4a). Moreover, significant correlations of DsrA with disease severity scores were also observed in the AsC/TI of CD and the DeC of UC patients (Supplementary Fig. 5). Given the regulating roles of l-cysteine and H_2_S in the mucosal inflammation^9,25^, our findings suggest possible microbial metabolic pathways that influence the host intestinal physiology and disease progression.

### Strain-specific alterations of *F. prausnitzii* in CD

**Fig. 5:**
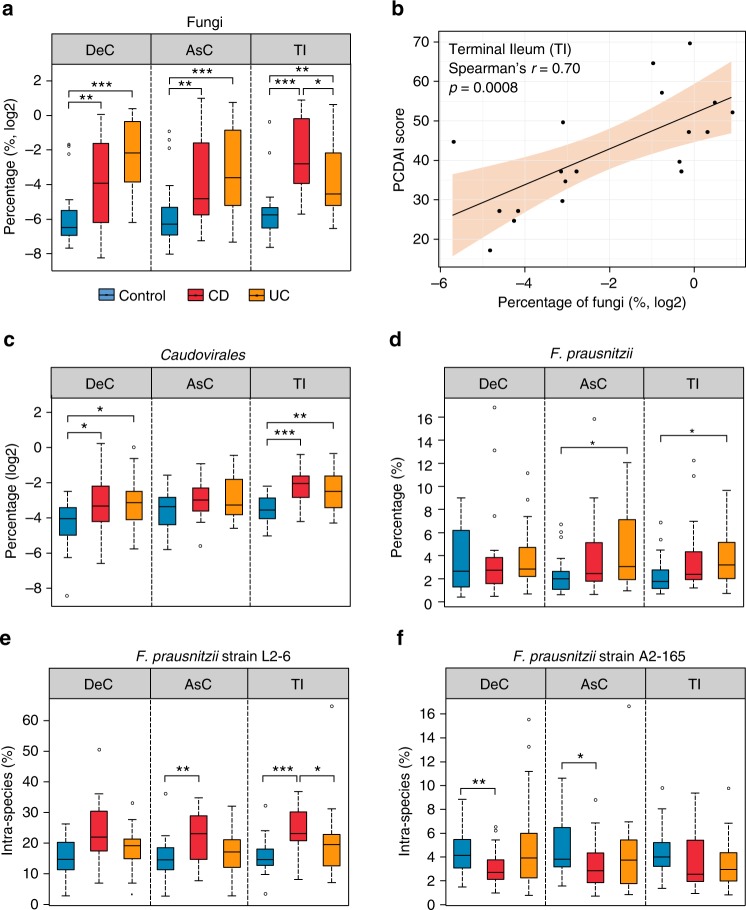
Alterations of MLI microbiome composition in pediatric IBD patients. **a** Relative abundance of Fungi in MLI microbiome. **b** Representative scatter plot between the fungal abundance and CD disease severity scores. **c** Relative abundance of *Caudovirales* in MLI microbiome. **d** Relative abundance of *F. prausnitzii* in MLI microbiome. **e**, **f** Intra-species relative abundance of *F. prausnitzii* strain L2–6 and A2–165. For the box plot, the bottom and top of the box are the first and third quartiles, respectively. The middle line represents the sample median. Whiskers are drawn from the ends of the IQR to the furthest observations within the 1.5 times the IQR range. Outliers >1.5 times the IQR are indicated with circle. *p < 0.05, **p < 0.01, ***p < 0.001

We identified 289 microbial species (189 ± 27 species per sample) with a minimum of 3 distinctive peptides from 4 different kingdoms (Bacteria, Fungi, Archaea, and Virus) (Supplementary Data 4). At the kingdom level, we found that Fungi was significantly increased in both CD and UC compared to control subjects (*p* < 0.01, Fig. 5a), which is in accordance with previous studies^26^. In addition, significant correlations were observed between fungal protein abundance and CD disease severity at both the AsC (Spearman’s *r* = 0.46, *p* = 0.03; not shown) and TI (Spearman’s *r* = 0.70, *p* = 0.0008; Fig. 5b). Linear discriminant analysis effect size (LEfSe)^27^ analysis identified 4 phyla, 7 classes, 11 orders, 11 families, 16 genera, and 16 species as differentially abundant in IBD compared to control in at least two intestinal regions (Supplementary Data 5 and Supplementary Fig. 6). All four differentially abundant phyla, namely Proteobacteria, Verrucomicrobia, Ascomycota, and Spirochetes, were increased in IBD compared to control. We found that the bacteriophage *Caudovirales* was significantly increased in IBD compared to controls (Fig. 5c), which is also in agreement with a previous virome metagenomics study^8^. The most significantly changed genera in IBD were *Bacteroides* and *Faecalibacterium*, which were decreased and increased, respectively, as compared to controls (Supplementary Fig. 6).

*Faecalibacterium prausnitzii*, the major species of *Faecalibacterium*, showed higher relative abundance in both UC and CD patients compared to control subjects; in particular, significant differences were observed in UC at the AsC and TI (Fig. 5d). This finding is in agreement with a previous 16 s rRNA gene sequencing study using the same sample type (i.e., AsC MLI aspirate) from pediatric IBD patients^9^. Similar findings have also been reported by Hansen et al.^28^ and Assa et al.^29^, who studied the microbiota compositions in biopsies collected from pediatric IBD patients using DNA sequencing and real-time PCR. However, controversial associations of *F. prausnitzii* with IBD, regarding their protective roles on gut barrier function, have been commonly reported^30^. To obtain a deeper understanding of *F. prausnitzii*’s role in IBD, we performed strain-resolved analyses and for the first time demonstrated that L2–6 was the dominant strain in the pediatric MLI samples and only this strain showed higher intra-species abundance in CD (Fig. 5e). Strain A2–165, which is more adept in producing butyrate compared to L2–6^31^, showed significantly lower intra-species abundance in CD compared to control subjects at the DeC and AsC (Fig. 5f). These results are in agreement with a previous study by Song et al.^31^ that demonstrated a subspecies-level dysbiosis of *F. prausnitzii* in the gut of atopic dermatitis patients with an increase of strain L2–6 and decrease of strain A2–165. In addition, pangenomic analysis of *F. prausnitzii* also reported that strain L2–6 uniquely encoded metabolic functions responsive to oxidative stress, such as *sufBC*^31^, enabling its competitive ability in stressful environment such as the gut of IBD patients. These findings provide evidence that the relationship between IBD and *F. prausnitzii* may be at the strain level instead of species level and its sub-species level dysbiosis may be related to their different competitive growth abilities in response to oxidative stress.

### Elevated MLI host defense response proteins in IBD

**Fig. 6:**
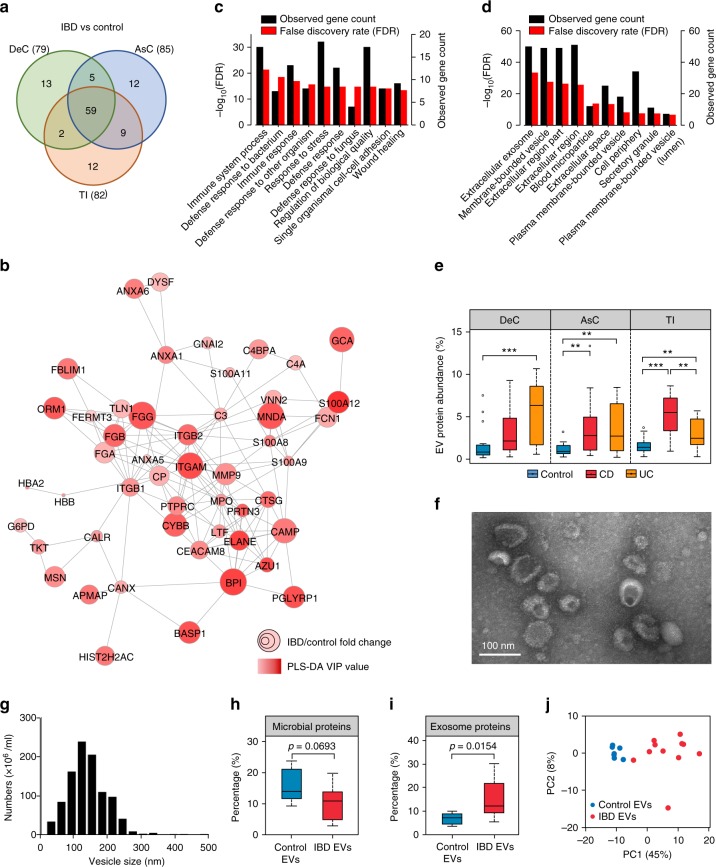
Alteration of MLI human proteins in pediatric IBD patients. **a** Numbers of differentially abundant proteins identified by PLS-DA with a threshold of VIP ≥ 2. The numbers in brackets indicate the total number of proteins identified for each intestinal region. **b** Protein–protein interaction network of the 59 differentially abundant MLI human proteins at all three intestinal regions in IBD. **c** Top biological processes and **d** top cellular components enriched by the differentially abundant MLI human proteins in IBD. **e** Percentage of EV related proteins in the MLI human proteomes. For the box plot, the bottom and top of the box are the first and third quartiles, respectively. The middle line represents the sample median. Whiskers are drawn from the ends of the IQR to the furthest observations within the 1.5 times the IQR range. Outliers > 1.5 times the IQR are indicated with circle. *p < 0.05, **p < 0.01, **p < 0.001. **f** Electron microscopy of isolated vesicles. **g** Nanoparticle tracking analysis of the isolated vesicle size distribution from DeC aspirates of patient HM537. **h** Percentage of microbial proteins in the isolated EV proteomes. **i** Percentage of exosome proteins matching to the top 100 proteins in ExoCarta database. **j** PCA score plot of the free EV human proteomes from patients with or without IBD

To identify the MLI human proteins that were differentially abundant in IBD compared to control or CD compared to UC, PLS-DA was conducted for each intestinal region. We identified 79, 85, and 82 IBD-related differentially abundant human proteins for the DeC (*Q*^2^ = 0.82), AsC (*Q*^2^ = 0.83), and TI (*Q*^2^ = 0.73), respectively, with a threshold of VIP ≥ 2 (Fig. 6a and Supplementary Data 6); 70, 71, and 96 disease subtype-related differentially abundant human proteins were identified for the DeC (Q^2^ = 0.57), AsC (Q^2^ = 0.48), and TI (Q^2^ = 0.39), respectively (Supplementary Data 7). Among the IBD-related differentially abundant proteins, 59 were common for all three intestinal regions, while there were only three disease subtype-related differentially abundant proteins (C-reactive protein, dual oxidase 2 and complement component 4 binding protein alpha chain) that were common for all regions (Supplementary Fig. 7). Among the 59 IBD-related differentially abundant human proteins, 58 were increased in IBD, with only the chloride anion exchanger (S26A3) decreased. Protein interaction networks of these 59 proteins yielded significant protein interactions (PPI enrichment *p* < 0.0001 as assessed using STRING^32^) (Fig. 6b). In the protein network, integrin alpha M (ITGAM; also known as CD11b), myeloperoxidase (MPO) and integrin beta-2 (ITGB2; also known as CD18) displayed the highest degree of interaction, connecting with 17, 17, and 15 proteins in the network, respectively (Fig. 6b), suggesting a “hub” role for these proteins.

Gene ontology enrichment analysis of the 59 differentially abundant human proteins demonstrated that the mostly enriched biological functions include immune and defense responses against bacterium or fungi (Fig. 6c and Supplementary Data 8). In addition, the majority of proteins were related to extracellular region (51/59) and/or extracellular exosome (50/59) (Fig. 6d). Interestingly, among the 3493 human proteins quantified in this study, 2340 proteins matched to the EVpedia database of EVs and 88 matched the top 100 EV proteins (defined by the frequency of identification in previous EV datasets)^33,34^. The relative abundance of EV proteins that correspond to the top 100 list demonstrated a significant increase in both CD and UC as compared to control, in particular in the TI of CD patients and the DeC of UC patients (*p* < 0.001; Fig. 6e), regions of the intestine which are most often inflamed for their respective subtypes. In addition, we performed transmission electron microscopy (TEM) examination of the pellet fraction of aspirate samples, which confirmed the presence of vesicles consistent in size with that of shed intestinal microvesicles or exosomes (Supplementary Fig. 8a, b).

To gain insights on the host-microbiome interaction, we performed co-occurrence analysis with all identified differentially abundant human proteins and microbial functions, and found that both human proteins and microbial functions showed substantial associations with each other (Supplementary Fig. 9). Microbial uracil-DNA glycosylase (UDG, COG1573) displayed the highest connections (*n* = 48) to human proteins acting as important microbial hub in connecting the host and microbiome nodes. UDG is an enzyme which participates in mutagenesis prevention, as part of the base-excision repair pathway and cleaves uracil, as well as oxidized cytosine derivatives generated under conditions with high oxidative stress^35^. As mentioned above, 13 out of the 54 significantly increased microbial COGs belonged to category L and V that were related to DNA damage/mismatch responses, indicating that in IBD, microbes are deploying a considerable number of defensive mechanisms against stressors derived from the host.

### Pediatric IBD MLI extracellular vesicle proteome changes

To further expand the EV protein findings, we isolated free EVs that were present in MLI supernatants from a cohort of 11 new-onset IBD participants and 7 controls (Supplementary Data 9) and performed the first proteomic characterization of intestinal EVs from pediatric IBD patients. Electron microscopy and nanoparticle tracking analysis (NTA) analysis showed that the isolated vesicles were membrane-enclosed and consistent in size with that of exosomes, shed microvesicles and bacterial outer membrane vesicles (OMV, Fig. 6f, g). No significant difference was observed between the mean sizes of EVs isolated from children with IBD and controls (Supplementary Fig. 8c). Proteomic analysis identified a total of 2744 proteins (1519 human proteins; 1225 microbial proteins). The intensity-based microbial abundance across all samples was 12.6% and was lower in the EVs isolated from IBD than those isolated from controls (Fig. 6h). Of those microbial peptide sequences which were taxonomically assigned at the phylum level, the majority were derived from Bacteroidetes (70.7%; genus *Bacteroides* and *Prevotella*) followed by Firmicutes (25.6%; *Faecalibacterium*) (Supplementary Fig. 10a). Phylum level comparative analyses indicated that Proteobacteria and Ascomycota were significantly increased in IBD EVs, coinciding with the abovementioned metaproteomic findings (Supplementary Fig. 10b–d). Of the top 100 proteins in ExoCarta, 81 were identified in this dataset and yielded a mean exosomal protein abundance of 12% across all samples with a significant increase observed in IBD (Fig. 6i). This observation may partly be due to the elevated MLI human proteins in IBD (Fig. 1c). We also identified exosomal markers implicated in exosome biogenesis including CD9, CD63, TSG101, ALIX/ PDCD6IP, FLOT1, FLOT2, Rab27A and the bacterial OMV marker OmpA (Supplementary Table 1)^36^. Moreover, proteins previously deemed as under-represented in EVs^36^ were either not identified or present at low abundances (Supplementary Table 1).

The PCA analysis of the free EV human proteins displays an obvious segregation between IBD and control subjects (Fig. 6j). A total of 64 proteins were identified as discriminant features that were either identified with PLS-DA (*Q*^2^ = 0.81; 37 proteins) or overrepresented in IBD or control (identified in >70% of IBD and <30% control or vice versa; 18 proteins). Comparing the 64 free EV differentially expressed human proteins with the aforementioned 59 differentially abundant MLI microbiome-associated human proteins yielded considerable overlap with 20 proteins identified in both datasets, such as MPO and bactericidal/permeability-increasing protein (BPI) (Supplementary Fig. 8d, e). Interestingly, these 64 EV proteins yielded significant enrichment of biological processes relating to innate immunity and host defense, including phagocytosis, immune response, defense response to fungus, and defense response to bacterium (Supplementary Data 10).

## Discussion

**Fig. 7:**
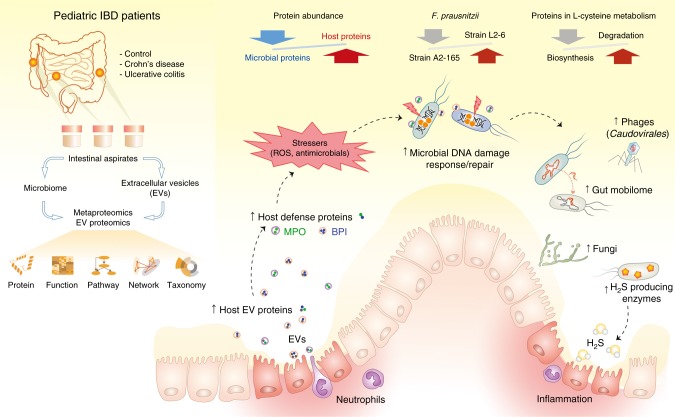
Graphic summary of the study design and main findings. Intestinal MLI aspirate samples were collected from three different intestinal regions from pediatric IBD patients or controls; the microbiomes and EVs were isolated and characterized using techniques including metaproteomics/proteomics approach. The differentially abundant human/microbial proteins, functions, pathways and microbial taxa were identified using multivariate statistical approaches. The intestinal MLI of IBD patients features an increased relative abundance of human proteins, in particular those EV-associated defense proteins, such as MPO and BPI, and a concomitant microbial dysbiosis at both functional and taxonomic levels

We, and others, have shown that aberrant microbiota–host crosstalk plays a central role in the pathogenesis of pediatric CD^9,37^, however, the mechanisms underlying this complex interplay in disease remains unclear. Through a metaproteomic approach, the current study identified both functional alterations of microbiome and the associated human protein signatures at the intestinal MLI in new-onset pediatric IBD patients, including those that have not been readily reported with metagenomics or metatranscriptomics^6,26,38^. Moreover, we demonstrated that the host defense proteins present in the intestinal EVs, including oxidative antimicrobial substrates, which would contribute to the excessive oxidative stress in the gut and thus abnormal host-microbiota interactions. A graphic summary of the study design and main findings is shown in Fig. 7. These findings provide new insights into the mechanisms of host–microbiome interaction and its role in the pathogenesis of IBD.

In evaluating the metaproteome of the intestinal MLI aspirate samples, we identified a substantial amount of human proteins. Interestingly a large proportion (67%) of these identified human proteins corresponded to proteins found in the EV protein databases^33^. EVs are membrane-bounded, protecting their cargo from proteases and nucleases which enables the delivery of bioactive molecules to neighboring cells, and thus EVs act as important mediators of long distance cell–cell communication and interaction^39^. In the intestinal lumen, EVs can be derived from multiple sources including intestinal epithelial/immune cells and microbial cells. Timár et al. demonstrated that EVs derived from neutrophils, an important component of intestinal mucosal immunity, had anti-bacterial activities which were dependant on ITGB2 function^40^. Accordingly, we found that ITGB2 (CD18) as well as ITGAM (CD11b, a marker that is consistently expressed on cell surface of neutrophils^41^), two subunits of αMβ2 integrin, were the most significantly increased MLI microbiome-associated human proteins in IBD and both also were hub proteins in the network of differentially abundant MLI human proteins identified in this study (Fig. 6b). The additional hub protein MPO, which is abundantly expressed in neutrophils and previously reported as a protein cargo of EVs released from neutrophils^40^, was also significantly increased in IBD. This elevated expression of MPO is also in agreement with previous observations from fecal samples^42–44^. Moreover, the proteomic characterization of isolated free EVs from intestinal aspirate samples also identified MPO as significantly increased in IBD compared to controls (Supplementary Fig. 8e). Given the ability of MPO to produce reactive oxidants such as hypohalous acids^45^, the host cell-derived EVs that carry increased amount of oxidative antimicrobials, such as MPO, may contribute to the elevated level of oxidative stress against microbes in the intestine of IBD patients.

Neutrophil infiltration of the intestinal mucosa is a common phenomenon in IBD. Li et al. have performed a metaproteomic characterization of the MLI proteins in adult CD and UC patients and found that the neutrophil-derived products such as human neutrophil peptide (HNP) 1–3 were significantly increased in both the MLI and biopsy samples^10^. In addition, other antimicrobial proteins derived from epithelium cells such as α-defensin 5, β-defensin 1 and 2, as well as transferrin and hepcidin that are involved in iron homeostasis, were among the most significantly increased proteins in the MLI adult patients^10^. In the current pediatric study, we also identified HNP3 and lactotransferrin (LTF) as increased in IBD compared to controls, however, we did not identify the other epithelium cell-derived ones. Instead, we identified neutrophil-derived proteins as the most significantly different MLI human proteins in IBD compared to control. This difference might be due to differences in the depth of metaproteomic measurement or sample processing procedures since we performed differential centrifugation to enrich the microbial cells and remove potential shedding intestinal cells or tissue debris. Therefore, human proteins directly binding to the microbial cells or carried by the human-derived vesicles were preferentially enriched and more readily identified in our study.

In addition to the MLI microbiome-associated human proteins, we also revealed marked functional alterations of microbiota in IBD patients. In particular, the microbial functions related to DNA damage repair and the microbial defense response were among the most significantly increased functions in IBD (Fig. 3a), which is in accordance with the observations of elevated host EV proteins, such as reactive oxidant-producing MPO, since reactive oxidants are known to mediate DNA damage^46^. Interestingly, we showed that the abundance of the UDG protein, an enzyme which cleaves uracils and repairs oxidative stress related DNA damage such as oxidized cytosine derivatives^35^, correlated to most of the host response proteins and aberrant microbial functions (Supplementary Fig. 9). These findings indicate that the elevated oxidative stress in the intestine of IBD patients might be a crucial driver for the aberrant host-microbiota interactions.

In addition, we found that the expression level of proteins related to transposase and prophage, namely mobilome, was increased in the microbiome of IBD patients compared to controls. The mobilome has been shown as a key factor in affecting the microbiome dynamics or adaptation in response to intestinal environmental changes^47^ and Diard et al. reported that the presence of inflammation and oxidative stress in the intestine can promote the induction of prophage lysogenic conversion^48^. Accordingly, we found a significant increase of proteins uniquely belonging to *Caudovirales* phages (Fig. 5c), in IBD compared to control, which is also in agreement with a previous virome metagenomics study^8^. In Prokaryotes, the CRISPR/Cas system acts as an important immune system against foreign nucleic acids, such as phage genome sequences^21,22^. Interestingly, we identified two CRISPR/Cas system related functions that were significantly increased in both CD and UC (Fig. 3a), further providing evidence for the bloom of bacteriophage in the microbiome of IBD patients. Given that these mobile gene elements (MGEs) are usually associated with the horizontal gene transfer (HGT) of antibiotic resistance determinants^49,50^, the findings in the current study may also suggest an increased susceptibility to antibiotic resistance of gut microbiota in patients with IBD.

We previously demonstrated by 16s rRNA gene sequencing an increase of H_2_S producers and decrease of butyrate-producers in pediatric IBD, which was associated with the down-regulation of mitochondrial H_2_S detoxifying proteins^9^. Here, we further revealed that microbial proteins with functions of l-cysteine desulfidase and dissimilatory sulfite reductase, two important pathways for H_2_S production, were increased in the MLI microbiome from pediatric IBD patients. In addition to H_2_S production, we found a significant increase of l-cysteine degradation (including the enzymes for H_2_S production) and decrease of l-cysteine biosynthesis in the intestinal microbiome of IBD patients. l-cysteine plays a vital role in regulating cellular redox potential through the synthesis of anti-oxidant glutathione (GSH)^51^. l-cysteine has also been shown to play important roles in maintaining the gut barrier functions^25,52^. Although it’s still unknown whether the alteration of l-cysteine metabolism is a cause or consequence of the elevated oxidative stress, our findings suggest that the dysregulated gut microbial L-cysteine utilization together with the elevated intestinal oxidative stressors may influence the development of mucosal inflammation in IBD.

In summary, we performed a large-scale gut metaproteomic study of a pediatric IBD inception cohort to assess both the intestinal microbiome and its associated human proteins. We characterized the functional activity of the MLI microbiota at the early stage of IBD and identified new host-microbiome signatures of pediatric IBD at the MLI, including the alterations of microbial mobilome and l-cysteine metabolism, both of which are indicative of elevated oxidative stress. In addition, we performed the first proteomic characterization of intestinal EVs from children with new-onset IBD and demonstrated that the host defense proteins present in the isolated EV samples, in particular the reactive oxidant-producing enzymes, may contribute to the increased oxidative stress in the intestine. The latter may lead to microbial defense responses and functional adaptations, which may initiate the gut microbial dysbiosis and in turn contribute to the development of mucosal inflammation. This study thereby provides valuable protein-level information for aiding in understanding the host–microbiome interactions underlying the development of IBD.

## Methods

### Subjects and MLI aspirate sample collections

Seventy-one pediatric patients (<18 years old) were included in this study. All eligible patients were undergoing clinically indicated endoscopic examination following their consultation to General Gastroenterology clinic for suspected IBD. None of the participants had ever been on any empiric IBD treatments. The most common reasons for colonoscopy were non-acute abdominal pain, reports of rectal bleeding, non-acute diarrhea and weight loss. Non-IBD controls had visually normal mucosa, histologically normal mucosal biopsies and normal imaging to definitively rule out IBD. The diagnosis of IBD was performed according to standard criteria following thorough clinical, endoscopic, histologic and radiological evaluation^53^. In total, 25 patients were diagnosed with CD, 22 patients were diagnosed with UC, and 24 patients were deemed non-IBD controls. The study and clinical sample collection protocol was approved by the Research Ethics Board of the Children’s Hospital of Eastern Ontario (CHEO), Ottawa, ON, Canada. All patients were newly diagnosed and naïve to treatment. Informed consent was obtained from all subjects. The following exclusion criteria were implemented: (1) presence of diabetes mellitus; (2) presence of infectious gastroenteritis within 2 months; (3) use of any antibiotics or probiotics within 4 weeks, or (4) irritable bowel syndrome. Clinical disease activity of CD was determined using the PCDAI (Pediatric Crohn’s Disease Activity Index)^54^ and of UC using the PUCAI (Pediatric Ulcerative Colitis Activity Index)^55^.

Intestinal MLI aspirate samples were collected from the DeC, AsC and/or TI during diagnostic colonoscopy for each participant. During colonoscopy, any loose fluid and debris was first aspirated and discarded. Thereafter, sterile water was flushed onto the mucosa to dislodge the mucus layer from the mucosal epithelial cells and the resulting mixture was aspirated into a container, which was immediately put on ice and transferred to the laboratory for processing.

### MLI aspirate sample processing for metaproteomic analysis

Aspirate samples were first centrifuged at 700 g, 4 °C for 5 min and the supernatant was transferred into a new tube for another centrifugation at 14,000 g, 4 °C for 20 min. This pellet fraction was then harvested for metaproteomics analysis. Protein extraction and trypsin digestion were performed according to our previously published procedure^15^. Briefly, proteins were extracted with protein lysis buffer containing 4 % (w/v) sodium dodecyl sulfate (SDS) and 6 M urea in 50 mM Tris-HCl buffer (pH 8.0). The protein lysates were then subjected to three ultrasonications (30 s each with 1 min interval on ice) using a Q125 Sonicator (Qsonica, LLC) with an amplitude of 25%. Remaining cell debris was removed through high-speed centrifugation at 16,000 g, 4 °C for 10 min. The resulting supernatant was then precipitated using 5-fold volume acidified acetone/ethanol buffer at −20 °C overnight. Proteins were pelleted by centrifugation at 16,000 g for 20 min at 4 °C, and washed with ice-cold acetone three times. The protein pellets were then re-suspended in 6 M urea, 50 mM ammonium bicarbonate buffer. For in-solution trypsin digestion, 50 µg of proteins for each sample were reduced and alkylated with 10 mM dithiothreitol and 20 mM iodoacetamide, respectively. One microgram of trypsin (Worthington Biochemical Corp., Lakewood, NJ) was then added for digestion at 37 °C overnight with agitation. The tryptic peptides were desalted with a 10-μm C18 column and the tryptic peptides were analyzed on a Q Exactive mass spectrometer (ThermoFisher Scientific Inc.). Briefly, peptides equivalent to 2 μg proteins were loaded and separated on an analytical column (75 μm × 50 cm) packed with reverse phase beads (1.9 μm; 120-Å pore size; Dr. Maisch GmbH, Ammerbuch, Germany) with 4 h gradient from 5 to 35% acetonitrile (v/v) at a flow rate of 200 nl/min. The instrument method consisted of one full MS scan from 300 to 1800 m/z followed by data-dependent MS/MS scan of the 12 most intense ions, a dynamic exclusion repeat count of 2, and repeat exclusion duration of 30 s. The MS data were exported as.RAW format, which were then processed with MetaPro-IQ bioinformatics workflow for peptide/protein identification and quantification^15^.

To ensure better coverage of the reference protein database, we generated an integrated human gut microbiome protein database, containing the proteins from (1) integrated gene catalog database (IGC) of human gut microbiome (containing 9,879,896 proteins)^56^; (2) NCBI reference sequence database of Virus (containing 275,423 proteins; downloaded at October 17, 2016 from ftp://ftp.ncbi.nlm.nih.gov/refseq/release/viral/); (3) 121,347 fungal protein sequences of 7 species that have been previously reported to present in pediatric IBD patients^26,57^, namely the proteomes of *Candida albicans*, *Candida parapsilosis*, *Cladosporium cladosporioides*, *Clavispora lusitaniae*, *Cyberlindnera jadinii*, *Kluyveromyces marxianus*, *Saccharomyces cerevisiae* (the proteome databases were downloaded at November 24, 2016 from UniprotKB: http://www.uniprot.org/proteomes/); (4) an in-house MLI gut microbial gene catalog database (containing 1,088,113 predicted gene sequences), generated from shotgun metagenomics sequencing for 28 MLI samples of a pediatric IBD sub-cohort (12 control, 7 CD and 9 UC) and with an approach described previously^15^. The two gene catalog databases were combined and the redundant sequences were removed using CD-HIT (-c 0.95 -M 0 -G 0 -aS 0.9 -g 1 -d 0 -T 0)^58^. Among the 1,088,113 predicted MLI genes, 75,425 genes (6.9%) were absent in the IGC database which therefore provides additional sequence information for microbial protein and peptide identification from MS data.

We used an iterative database search strategy, namely MetaPro-IQ, that has been implemented as an integrated analysis platform (MetaLab)^15,59^, to increase the sensitivity of the large database search. Iterative database search strategies have previously been shown to improve the sensitivity of peptide identification using large databases and enable comparable performance to a matched metagenome database search strategy^15,60^. Briefly, each of the raw files was searched against the above generated integrated microbiome protein database. All matched protein sequences were extracted from each run and combined to generate a refined and size-reduced database. This refined database was then concatenated with the human protein database (downloaded from UniprotKB on 24 December 2016) for a target-decoy database search (at false discovery rate of 0.01) and label-free quantification using the MaxLFQ algorithm^61^. Both razor and unique peptides were used for protein quantification, and the minimum ratio count was set as 1. An alignment retention time window of 20 min and match time window of 5 min were applied to match the same accurate masses between different runs. Proteins identified by the same set or a subset of peptides were grouped together as one protein group. A protein group was considered as human or microbiome protein group only when all the majority proteins were from human or microbiome proteome database, respectively. Similarly, a peptide sequence was considered as being originated from human or microbiome only when all the matched proteins were from human or microbiome proteome database, respectively.

### Free EV isolation, protein extraction and proteomics analysis

Debris and microbial cells in the MLI aspirate samples were depleted as described above. The resulting debris- and bacteria-depleted supernatant was filtered through a 0.45 μm syringe driven filter and the resulting supernatant was stored at −80 °C. EV isolation was performed according to the protocol used by Mitsuhashi et al. with modifications^62^, although it should be noted that contaminating soluble proteins may be co-isolated with the EVs. Briefly, the supernatant was subjected to ultracentrifugation at 100,000 g for 70 min at 4 °C. The EV pellet was washed with PBS buffer and subjected to another round of ultracentrifugation at 100,000 g for 70 min. A portion of the resuspended pellet was lysed in 4% (w/v) SDS, 50 mM Tris-HCl (pH 7.8). A total of 25 μg of proteins was digested by filter-aided sample preparation (FASP) procedure^63^. The resulting tryptic peptides were desalted, analyzed, and data processed as described above.

### Characterization of isolated free EVs

Size distribution of the isolated EVs was examined with NTA using the ZetaView nanoparticle tracking analyzer (Particle Metrix GmbH, Germany) using a shutter value of 40 and sensitivity of 85. Prior to each session, the size of standard silica beads was measured. The EV preparations were diluted 1:25,000–1: 250,000 in PBS to yield a concentration consistent with the accuracy range of the instrument. Measurement data obtained from the ZetaView output was analyzed in Excel and plotted in Prism 7.0; EV sizes were binned by 30 nm.

The morphology of EVs was characterized with TEM. Briefly, the differential centrifugation pellets (14,000 g and 100,000 g) were firstly fixed in 2.5% glutaraldehyde in a 0.1 M sodium cacodylate buffer. For negative staining, the pellets were suspended and a drop of suspension was added on to a formvar grid. When the suspension had partly dried, the grid was washed by adding a drop of distilled water and touching it three times to the surface of water droplet. Excess water was removed by touching the grid to a filter paper. After drying, a drop of 2% uranyl acetate stain was added to the grid and incubated for 10 s before removing the excess stain and drying at room temperature for imaging. For sectioning, the glutaraldehyde fixative was removed from the pellet and replaced with 2% OsO_4_ in 0.1 M sodium cacodylate buffer. The OsO_4_ fixative was replaced with 0.1 M sodium cacodylate before dehydrating in an increasing series of alcohol. The most concentrated alcohol was replaced with acetone. The material was penetrated by a growing series of Araldite diluted in acetone, and finally embedded in Araldite (Huntsman Advanced Materials LLC, United States). Ultrathin sections (80 nm) were prepared using an Ultracut Leica UC6 ultramicrotome (Leica Microsystems, Germany) and placed onto a copper grids coated with formvar film. Sections were stained with uranyl acetate and lead citrate solutions and examined with a transmission electron microscope (JEOL JEM 1230, Japan).

### Microbial taxonomic analysis

All identified peptide sequences were subjected for taxonomic analysis using lowest common ancestor (LCA) algorithm implemented in MetaLab^59^. The calculation of LCA was performed according to the principles implemented in Unipept with modifications^64^. In the current study, NCBI taxonomic nodes containing words “uncultured”, “unspecified”, and “undetermined” were excluded, however species nodes with numbers in their name were kept for the LCA calculation. Two missed cleavages of peptides were allowed, which is equivalent to “Advanced missed cleavage handling” options in Unipet web application^65^. To ensure confident taxonomic identification, taxa with ≥ 3 distinctive peptides were used further analysis. The sum intensity of all distinctive peptides for a taxon was used for calculating relative abundance of the taxon within a specific rank level. Strain-resolved analysis of *Faecalibacterium prausnitzii* was performed as previously described^13^. Briefly, strain-distinctive peptides of *Faecalibacterium prausnitzii* were identified by comparing *F. prausnitzii* species distinctive peptides with all the in silico tryptic peptides (with maximum of 2 missed cleavages) of *F. prausnitzii* strain databases available in UniprotKB proteome database (downloaded on October 25, 2016). A total of five strain proteomes of *F. prausnitzii* were obtained, including UP000006028 for strain KLE1255, UP000004619 for strain A2–165, UP000007059 for strain SL3/3, UP000008804 for strain L2–6, and UP000005945 for strain M21/2. Intra-species abundance of each strain was then calculated as the percentage of distinctive peptide intensity out of the total intensity of all strain-distinctive peptides.

### Microbial function and pathway analysis

All quantified microbial protein sequences were annotated with the COG database (version 2014) as previously described^15^. KEGG ortholog (KO) annotation of protein sequences was conducted with the GhostKOALA web application^66^. Taxonomy assignment of the proteins were performed using MEGAN 6^67^. The taxonomy of a protein group was assigned with the lowest common ancestor (LCA) of all proteins within that protein group.

### Protein–protein interaction and pathway enrichment analysis

Protein–protein interaction (PPI) and pathway enrichment analysis were performed with STRING (version 10.5)^32^. The protein interaction networks were exported from STRING and visualized using Cytoscape software (version 3.4.0).

### Statistical and multivariate data analysis

The LFQ intensity of quantified protein group was log_10_-transformed and used for statistical analysis. Univariate statistical difference between each pair of groups was assessed by Wilcoxon rank-sum test, except when indicated otherwise. For multivariate statistical analysis, only those protein groups with valid LFQ intensity values in ≥ 50% of the samples were used. Missing values were imputed with the K-nearest neighbors method. PCA was performed in MATLAB (The MathWorks Inc.). Hierarchical clustering analysis and heatmap plotting was performed with R (https://www.r-project.org/). Partial least squares discriminant analysis (PLS-DA) was performed in MetaboAnalyst 3.0^68^ and important features were selected with a threshold of VIP (Variable Importance in Projection) ≥2.0.

## Electronic supplementary material

Supplementary Information

Peer Review File

Description of Additional Supplementary Files

Supplementary Data 1

Supplementary Data 2

Supplementary Data 3

Supplementary Data 4

Supplementary Data 5

Supplementary Data 6

Supplementary Data 7

Supplementary Data 8

Supplementary Data 9

Supplementary Data 10

## Data Availability

All MS proteomics data that support the findings of this study have been deposited to the ProteomeXchange Consortium (http://www.proteomexchange.org) with the dataset identifiers PXD007819 [http://proteomecentral.proteomexchange.org/cgi/GetDataset?ID = PXD007819] and PXD007959 [http://proteomecentral.proteomexchange.org/cgi/GetDataset?ID = PXD007959].
