# Supplementary material for: Metaproteomics reveals associations between microbiome and intestinal extracellular vesicle proteins in pediatric inflammatory bowel disease
Source: Nat Commun. 2018 Jul 20;9:2873. doi: 10.1038/s41467-018-05357-4 (PMC6054643; doi:10.1038/s41467-018-05357-4)
Supplement: Supplementary file 1 — Supplementary Information [file 41467_2018_5357_MOESM1_ESM.pdf]

Supplementary Information:

Metaproteomics reveals associations between microbiome and intestinal extracellular vesicle proteins in pediatric inflammatory bowel disease

Zhang *et al.*

Supplementary Note 1

To identify biogeographic proteins that show differential expression across the gastrointestinal tract, we used paired-sample T-tests to compare DeC vs. AsC, DeC vs. TI and AsC vs. TI for individuals in the control group. In total, we identified 226 protein groups that were significantly differentially abundant (FDR < 0.05) in at least one comparison (Supplementary Data 2). Among the 226 biogeographic proteins, 50 human proteins and 7 microbial proteins were significantly different in at least two comparisons, among which 49/50 human proteins and 3/7 microbial proteins showed higher expression levels in terminal ileum than the colon (Supplementary Fig. 1). In agreement with a previous adult cohort study[1], we also showed that elastase subfamily proteins and meprin A subunit proteins MEP1A/MEP1B exhibited decreasing expression levels from proximal to distal colon. Thirty out of the 49 previously identified adult intestinal biogeographic human proteins were also identified in this study (Supplementary Data 2). Among the 7 biogeographic microbial proteins, 5 were from Firmicutes, 1 from Bacteroidetes and the other from Actinobacteria. The protein from Bacteroidetes showed significantly lower expression level in TI compared to both the DeC and AsC, which is in agreement with previous phylogenetic observations in healthy adults[2].

Supplementary Tables

Supplementary Table 1 Exosomal markers, bacterial OMV marker OmpA and proteins expected to be absent of under-represented in EVs/exosomes and their associated mean LFQ and MS/MS count.

Types	Protein ID	Gene name	MS/MS Count	Mean LFQ intensity[b]	Mean LFQ/ CANX LFQ	Mean LFQ/ HSP90B1 LFQ	Mean LFQ/ OmpA LFQ
Known exosomal markers[a]	A6NNI4	CD9	239	3.85E8	207.86	36.70	7.65
	F8VNT9	CD63	256	1.22E8	65.86	11.63	2.42
	F5H442	TSG101	31	1.96E7	10.61	1.87	0.39
	Q8WUM4	PDCD6IP	664	4.66E8	251.87	44.47	9.27
	H3BN55	RAB27A	65	1.80E7	9.74	1.72	0.36
	O75955	FLOT1	17	1.13E7	6.12	1.08	0.23
	J3QLD9	FLOT2	28	1.67E7	9.04	1.60	0.33
Known under-represented proteins*	Q08379	GOLGA2	0	0			
	P08574	CYC1	0	0			
	P27824	CANX	6	1.85E6			
	P14625	HSP90B1	16	1.05E7			
OmpA	MH0032_GL0030062	N/A	2	1.61E6			
	MH0379_GL0175772	N/A	3	1.90E6			
	MH0064_GL0025357	N/A	18	2.15E7			
	MH0091_GL0024585	N/A	31	2.19E7			
	MH0196_GL0181180	N/A	1	4.13E4			
	V1.FI17_GL0222498	N/A	1	2.38E6			
	V1.FI18_GL0094903	N/A	5	9.88E5			

[a] according to the minimal experimental requirements for definition of extracellular vesicles proposed by Lötvall *et al.*[3]

[b] For the Mean LFQ intensity calculation a value of zero was assigned for those proteins which were not detected by MS.

Supplementary Figures

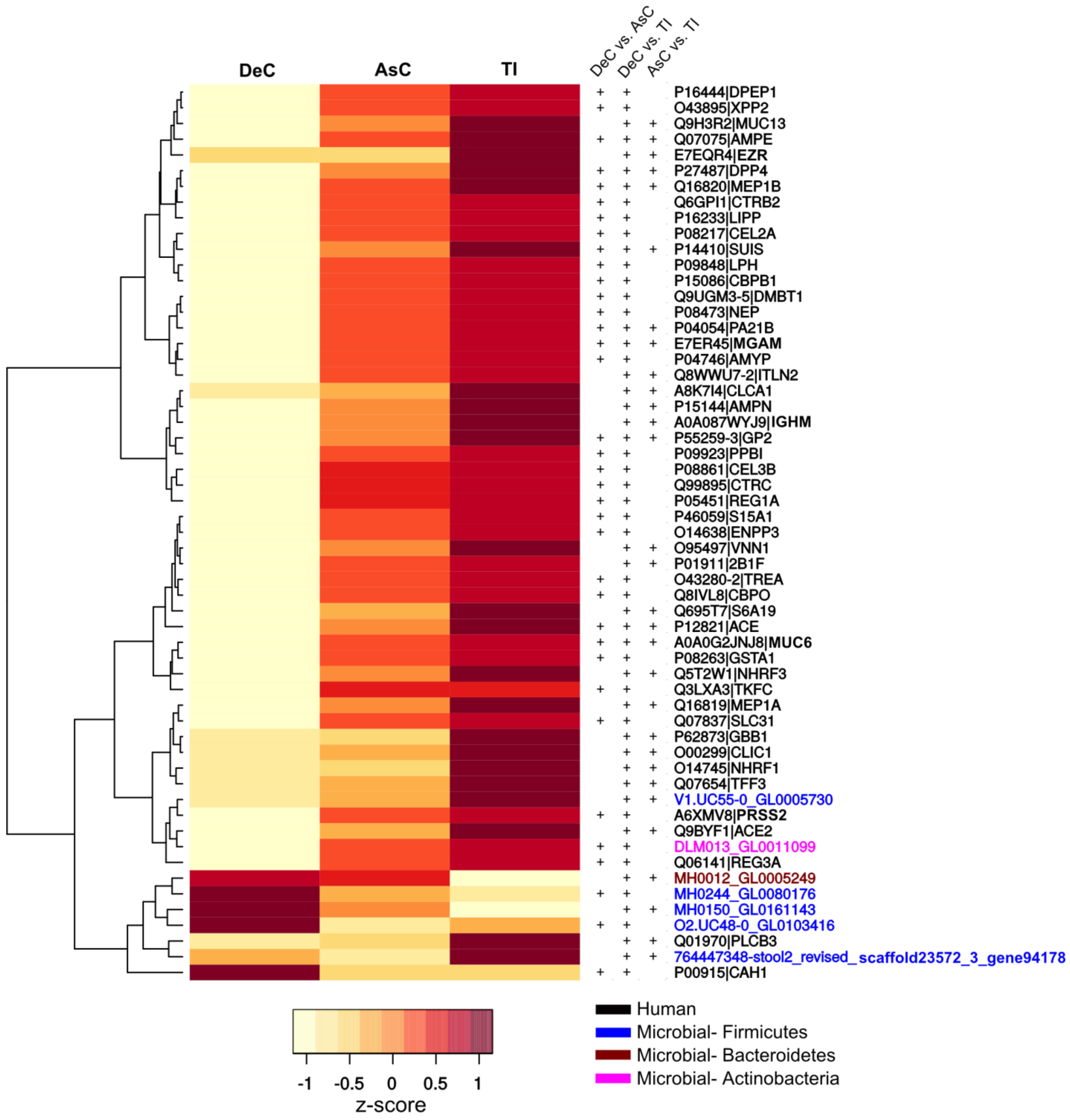

Supplementary Fig. 1 Intestinal biogeographic proteins at the mucosal-luminal interface of pediatric individuals. To avoid bias introduced by the presence of inflammation, only control subjects were included in this biogeographic analysis. Paired sample T-tests were used for evaluating the statistical significance between the DeC and AsC, DeC and TI or AsC and TI. Proteins significant in at least two comparisons (FDR < 0.05) are shown. The colors indicate the z-score and log10-transformed average LFQ intensity for each intestinal region. Row clustering was calculated with the Euclidean distance. Protein IDs were indicated in the right panel and details are presented in Supplementary Table 11. +, paired-sample T-test FDR < 0.05.

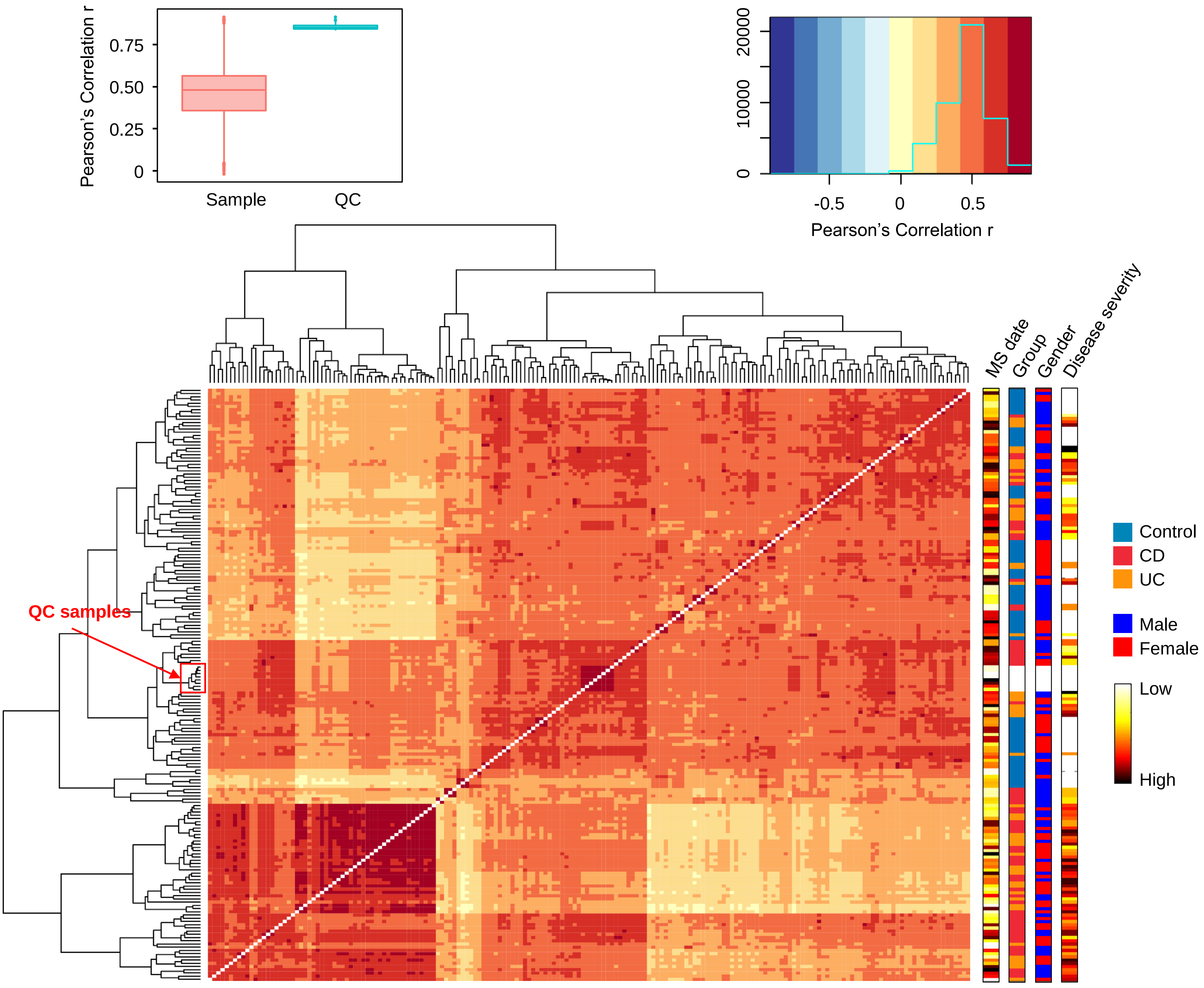

Supplementary Fig. 2 Heatmap of sample-sample correlation coefficient calculated with quantified MLI metaproteomes across all samples (176 microbiome and 8 QC samples). Both row and column clustering was calculated with the Euclidean distance. MS date, disease group, gender and severity for each sample were indicated. The distribution of Pearson's Correlation r in across all samples (upper-right) or Samples/QC (upper-left) are indicated.

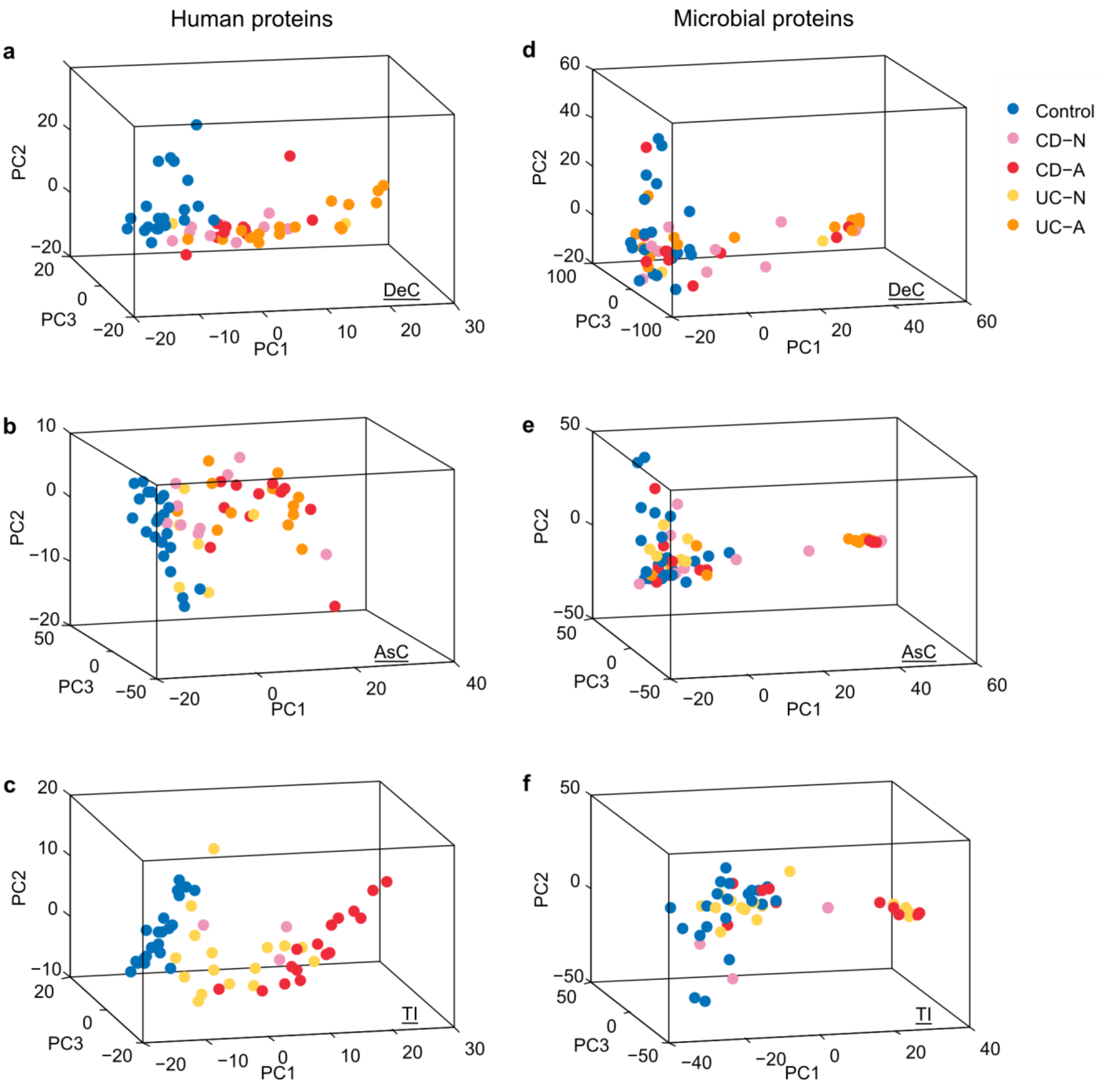

Supplementary Fig. 3 MLI host and microbiota proteome at different intestinal region of pediatric IBD patients. (a-c) PCA score plot of MLI human-derived proteins at DeC, AsC and TI, respectively; (d-f) PCA score plot of MLI microbiome-derived proteins at DeC, AsC and TI, respectively.

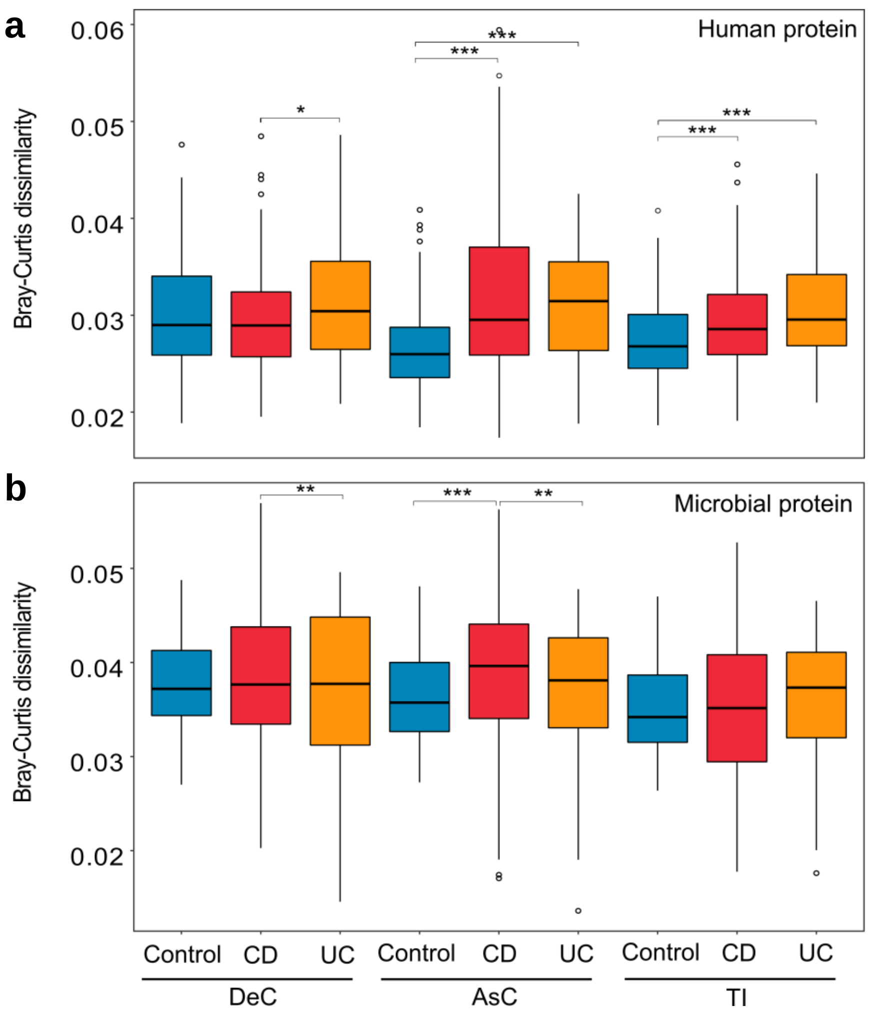

Supplementary Fig. 4 Within-group pairwise Bray-Curtis dissimilarity of quantified human (a) and microbial proteins (b) in pediatric IBD. Statistical significance of differences was evaluated with one-way ANOVA for each intestinal region. For the box plot, the bottom and top of the box are the first and third quartiles, respectively. The middle line represents the sample median. Whiskers are drawn from the ends of the interquartile range (IQR) to the furthest observations within 1.5 times the IQR range. Outliers >1.5 times the IQR are indicated with circle. *, $p < 0.05$; **, $p < 0.01$; ***, $p < 0.001$.

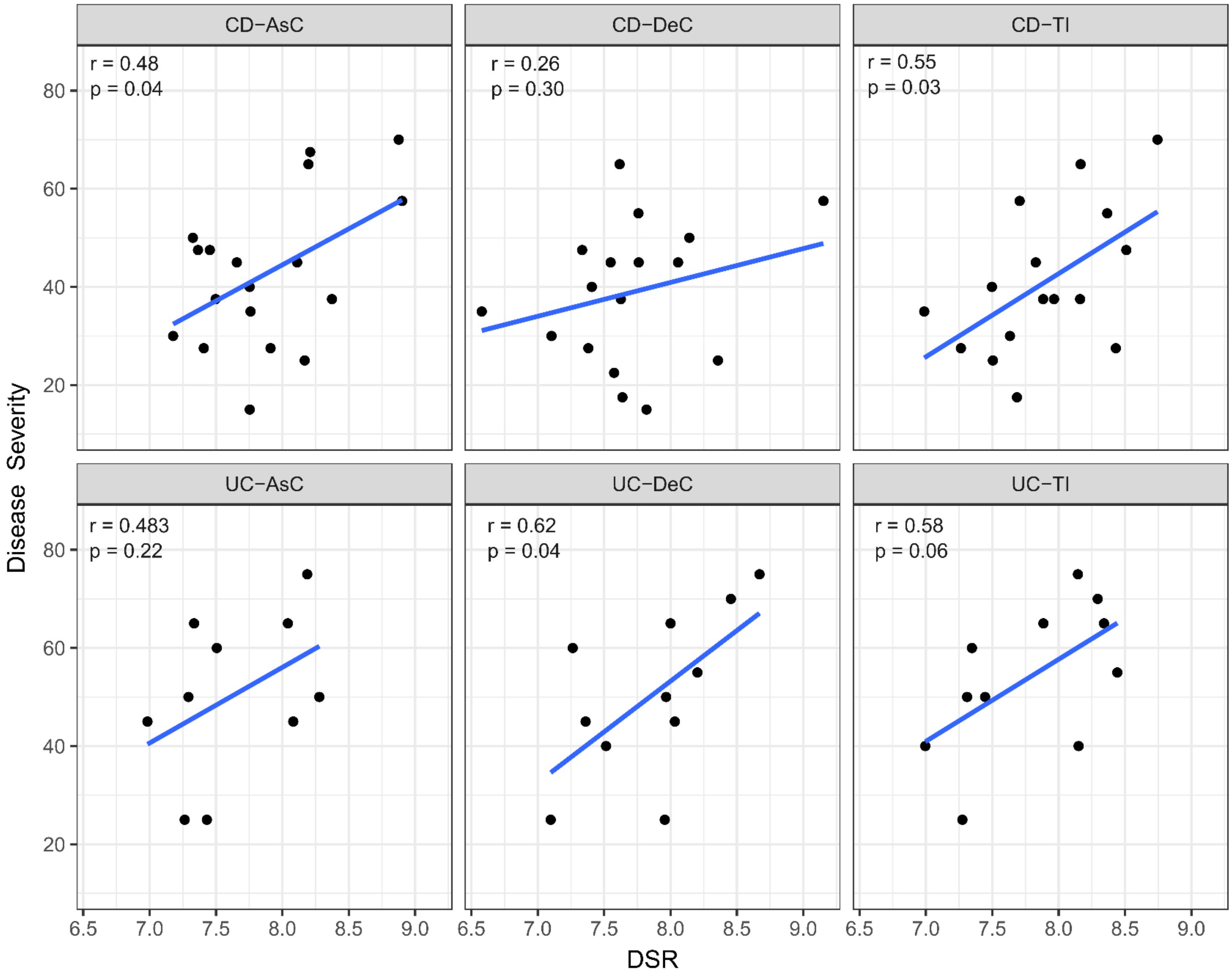

Supplementary Fig. 5 Scatter plots of the correlation between dissimilatory sulfite reductase with disease severity. Pearson's correlation r and p were indicated. PCDAI and PUCAI disease severity scores were used for CD and UC respectively. DSR, dissimilatory sulfite reductase.

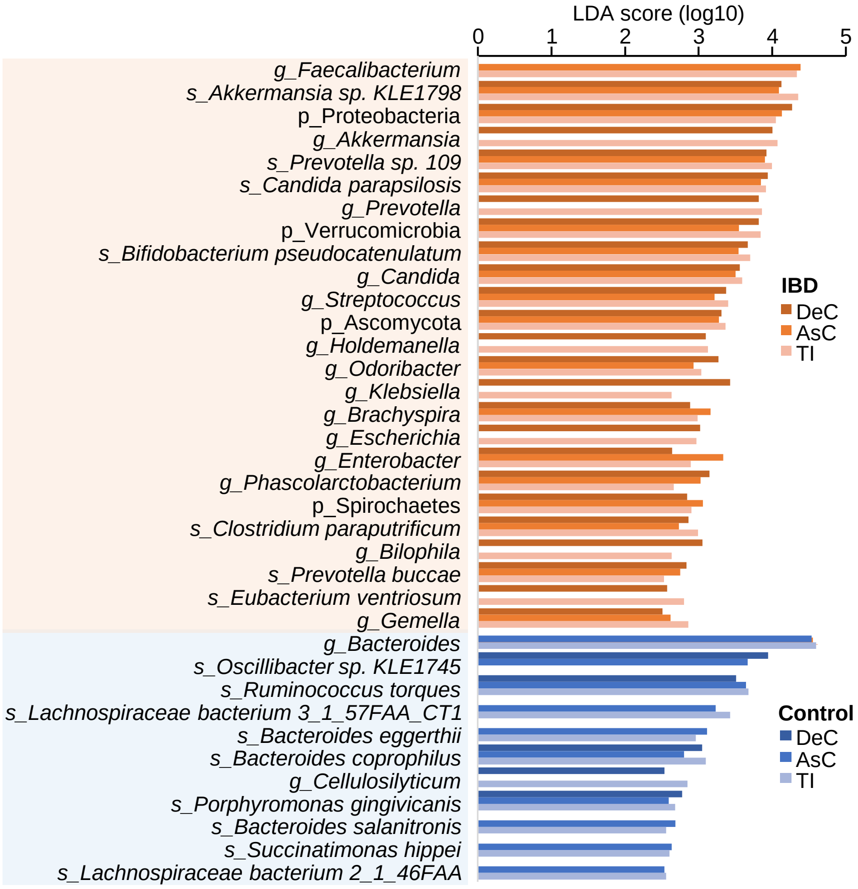

Supplementary Fig. 6 Taxonomic alterations of MLI microbiome in pediatric IBD patients. Differentially abundant phyla, genera and species identified using LEfSe analysis were shown.

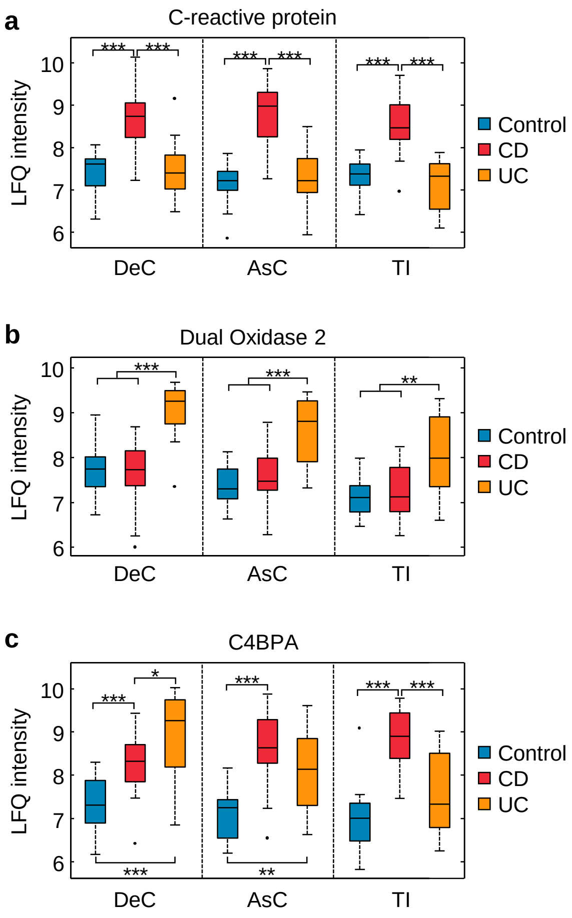

Supplementary Fig. 7 MLI human proteins segregate CD from UC. LFQ intensity of C-reactive protein (a), dual oxidase 2 (b) and complement component 4 binding protein alpha (C4BPA) (c) in MLI samples of pediatric CD or UC patients. Wilcoxon test was performed for each intestinal region individually. * $p < 0.05$, ** $p < 0.01$, ** $p < 0.001$. For the box plot, the bottom and top of the box are the first and third quartiles, respectively. The middle line represents the sample median. Whiskers are drawn from the ends of the IQR to the furthest observations within the 1.5 times the IQR range. Outliers >1.5 times the IQR are indicated with circle.

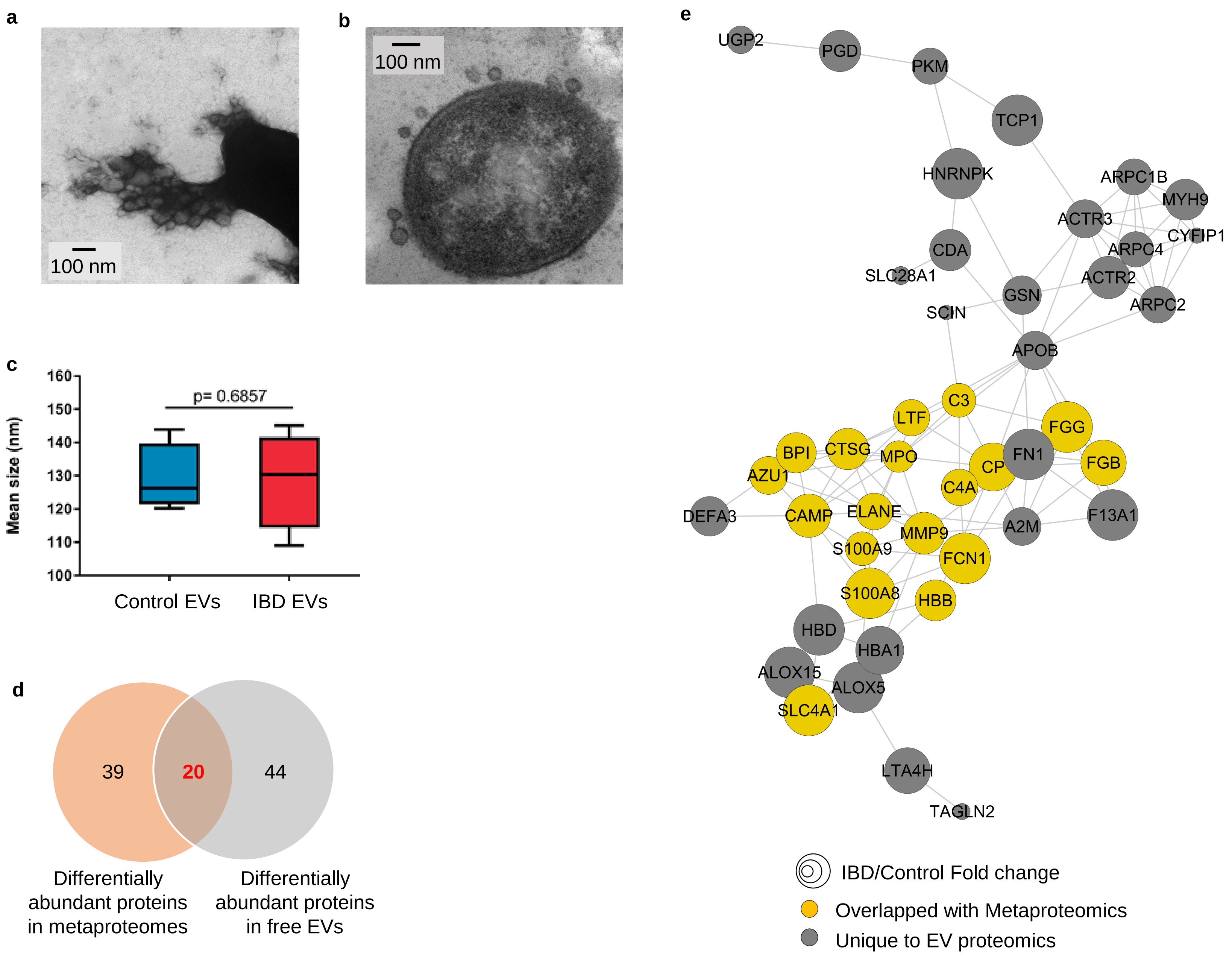

Supplementary Fig. 8 Characterization of the extracellular vesicle proteins in the MLI samples. (a) and (b) show the microbial-associated vesicles examined with TEM on negatively stained samples and sectioned, negatively stained samples, respectively. (c) Size distribution of EVs isolated from patients with and without IBD as measured by Nanoparticle tracking analysis. (d) Overlap between the 59 microbial-associated differentially abundant human proteins and those identified from free EV proteomics data set. (e) Protein-protein interaction network of the differentially expressed proteins in isolated free EVs. Node size indicates the log2-transformed fold change of relative protein expressions in IBD vs control.

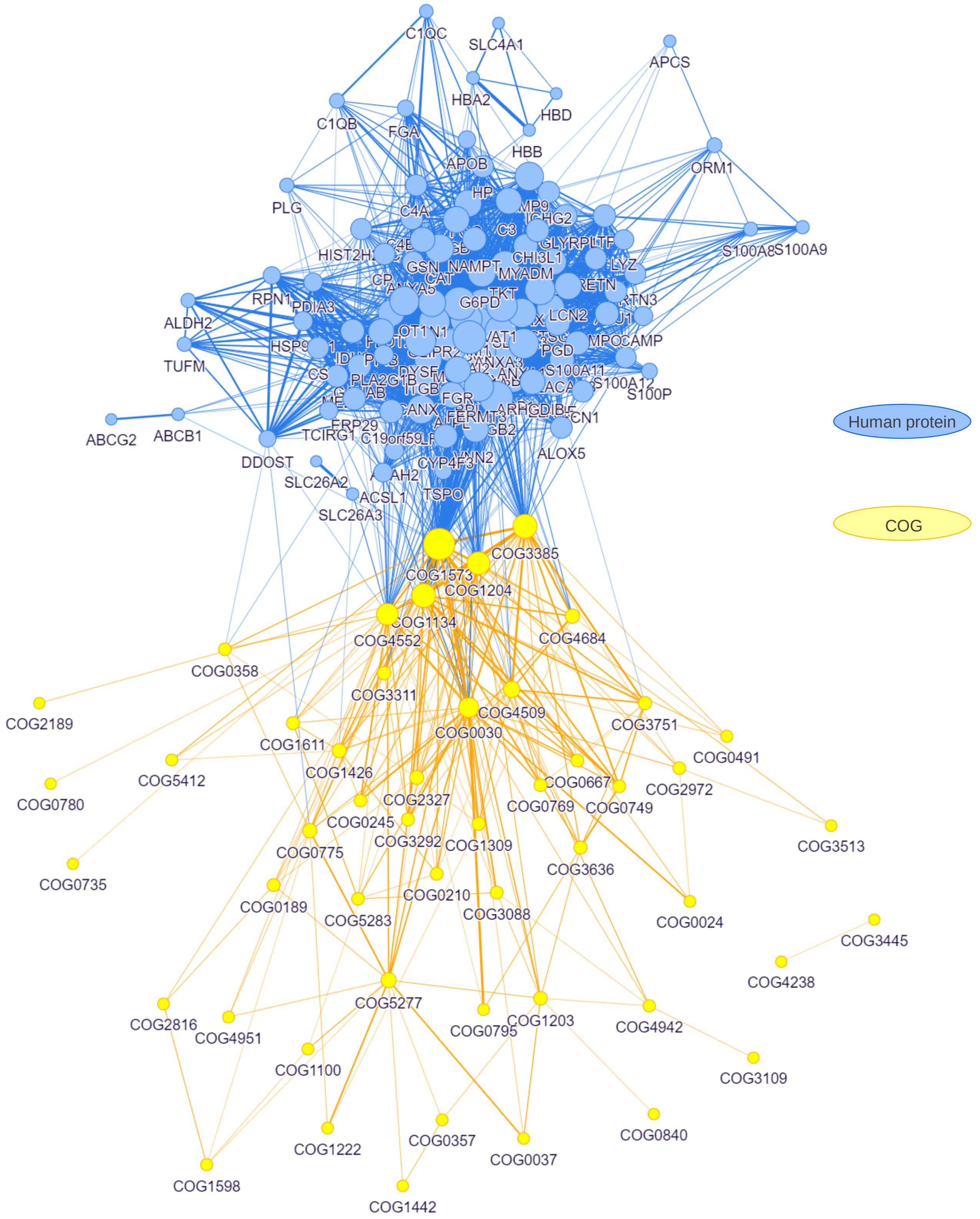

Supplementary Fig. 9 Network of co-occurrence of MLI human proteins and microbial functions that were differentially abundant in IBD. Spearman's correlation was used for constructing the network and the edge indicates a Spearman's r value of > 0.7 or < -0.7 and p < 0.05. The size of nodes is proportional to the number of connections and the thickness of edges indicates the Spearman’s r values. Yellow color indicates microbial COG while blue indicates MLI human proteins.

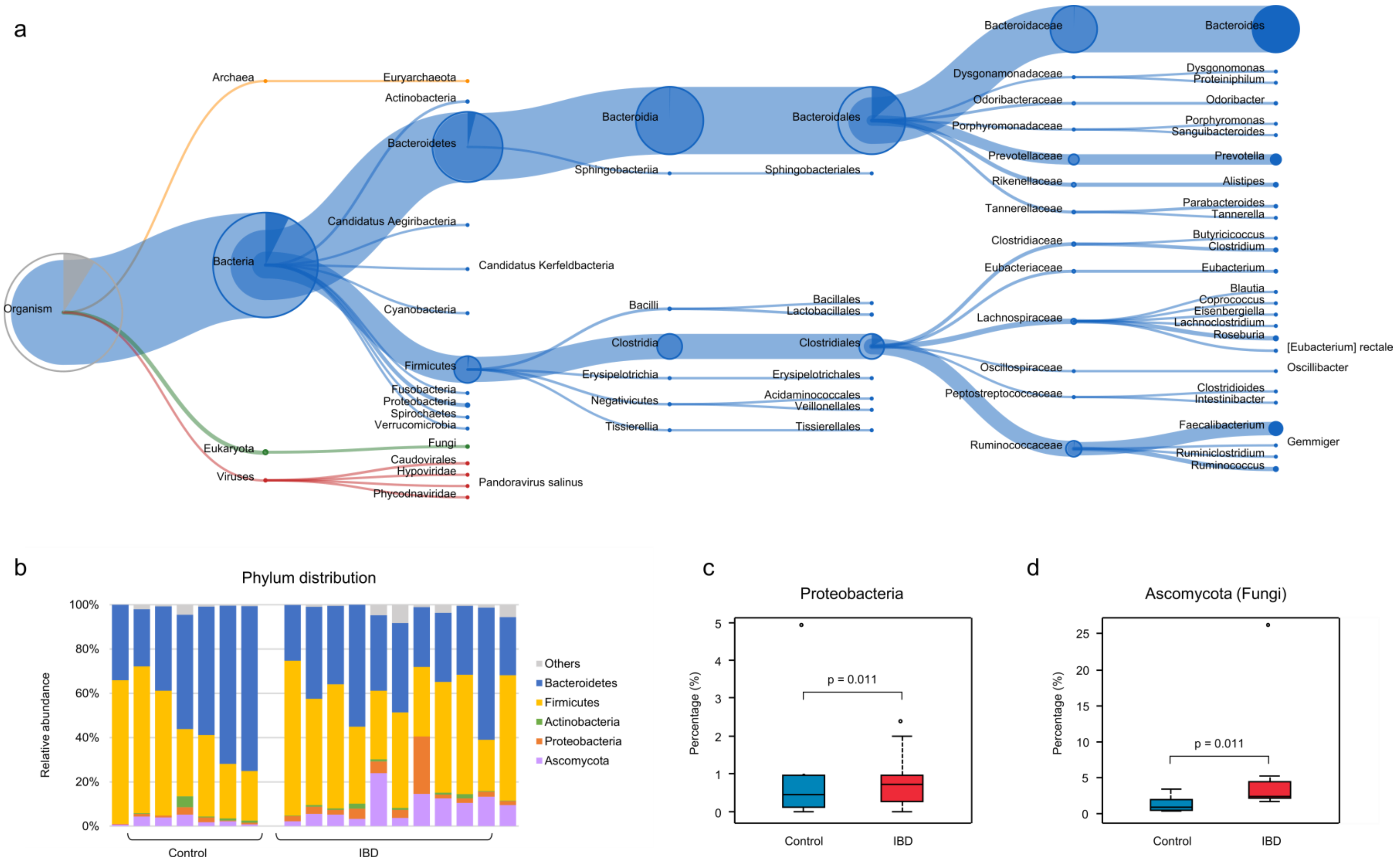

Supplementary Fig. 10 Taxonomic analysis of identified microbial peptides in EVs isolated from the intestinal MLI. (a) Tree view of the microbial taxa with all identified microbial peptide sequences and lowest-common ancestor (LCA) algorithm. The thickness of the branch and size of the node corresponds to the number of peptides associated with that node or any of its children. Pie charts in the node indicate the proportion of peptides that can be further assigned with a lower level taxonomy. (b) Stacked column chart showing the relative abundance of each phylum. Peptide intensity was considered in this plot. (c – d) Percentage of Proteobacteria and Ascomycota. Statistical significance was evaluated using Mann–Whitney U test. For the box plot, the bottom and top of the box are the first and third quartiles, respectively. The middle line represents the sample median. Whiskers are drawn from the ends of the IQR to the furthest observations within 1.5 times the IQR range. Outliers >1.5 times the IQR are indicated with circle.

References

1. Li, X., *et al.* A metaproteomic approach to study human-microbial ecosystems at the mucosal luminal interface. *PLoS One* **6**, e26542 (2011).

2. Zoetendal, E.G., *et al.* The human small intestinal microbiota is driven by rapid uptake and conversion of simple carbohydrates. *The ISME journal* **6**, 1415-1426 (2012).

3. Lotvall, J., *et al.* Minimal experimental requirements for definition of extracellular vesicles and their functions: a position statement from the International Society for Extracellular Vesicles. *Journal of extracellular vesicles* **3**, 26913 (2014).
