# Supplementary material for: Metaproteomics reveals associations between microbiome and intestinal extracellular vesicle proteins in pediatric inflammatory bowel disease
Source: Nat Commun. 2018 Jul 20;9:2873. doi: 10.1038/s41467-018-05357-4 (PMC6054643; doi:10.1038/s41467-018-05357-4)
Supplement: Supplementary file 3 — Description of Additional Supplementary Files [file 41467_2018_5357_MOESM3_ESM.pdf]

Description of Additional Supplementary Files

File Name: Supplementary Data 1
Description: Patient Characteristics of metaproteomic samples

File Name: Supplementary Data 2
Description: Mucosal-luminal interface biogeographic site-specific proteins

File Name: Supplementary Data 3
Description: Differentially abundant microbial COGs in IBD

File Name: Supplementary Data 4
Description: All identified microbial taxa in MLI samples

File Name: Supplementary Data 5
Description: Differentially abundant microbial taxa in IBD

File Name: Supplementary Data 6
Description: Differentially abundant MLI human proteins in IBD

File Name: Supplementary Data 7
Description: Differentially abundant MLI human proteins between IBD subtypes

File Name: Supplementary Data 8
Description: Gene ontology enrichment of differentially abundant MLI human proteins in IBD

File Name: Supplementary Data 9
Description: Patient Characteristics of EV proteomic samples

File Name: Supplementary Data 10
Description: Gene ontology enrichment of differentially abundant EV human proteins in IBD
