# Supplementary material for: Metaproteomics reveals associations between microbiome and intestinal extracellular vesicle proteins in pediatric inflammatory bowel disease
Source: Nat Commun. 2018 Jul 20;9:2873. doi: 10.1038/s41467-018-05357-4 (PMC6054643; doi:10.1038/s41467-018-05357-4)
Supplement: Supplementary file 2 — Peer Review File [file 41467_2018_5357_MOESM2_ESM.pdf]

Reviewers' comments:

Reviewer #1 (Remarks to the Author):

This study addresses an important and timely question regarding the growing role of wildfire in influencing permafrost thaw in peatlands and the expansion of thermokarst bogs. Understanding and quantifying the effects of wildfire on permafrost thaw is essential for predicting future greenhouse gas emissions and feedbacks to global climate change, and has not yet been well-documented. This paper will be of great interest to permafrost scientists, ecologists, climate scientists, and the broader community interested in global change. The authors clearly demonstrate the temporal trajectory of permafrost thaw and recovery in peat plateaus after wildfire through field sampling, and use remote sensing to document and scale up the increased rates of thermokarst bog expansion after fire. The study is well-designed and the presentation is polished. I have only a few minor comments for the authors to consider.

line 14: Perhaps specify that “effects of wildfire *in peat plateaus* were found to last for 30 years…”. As currently written, the sentence appears to suggest that effects of fire on permafrost overall only last for 30 years, contrary to what is stated in the discussion, that the effects of fire on permafrost via thermokarst bog expansion is considered irreversible at relevant timescales.

figure 3/lines 149-161: Interesting how clear and consistent these changes in permafrost and vegetation are over time since fire. This timeline of degradation/recovery is consistent with the theoretical work presented in Jafarov et al 2013. Their thermal model simulations showed permafrost recovery in Alaskan peatlands approx. 30 years after fire as well, and also found only minor impacts of fire severity in deep organic soils.

lines 187-188: Do you have any information regarding the depth of the taliks? It’d be interesting to know how deeply a talik could form and allow subsequent permafrost recovery in the current climatic conditions.

lines 303-305: You might need to explain somewhere in the discussion why the permafrost thaw in peat plateau centers is reversible but the permafrost thaw through thermokarst bog development at plateau edges is not reversible (the role of water and vegetation in soil thermal regimes?). Also, I’m curious if you found that the thawing of permafrost in plateau centers after fire resulted in the initiation of any small thermokarst depressions that subsequently stabilized. Brown et al 2015 noted that ice-rich permafrost thawed deeply in an Alaskan peatland after an old fire, but eventually refroze, leaving behind thermokarst microtopography.

References

Brown, D. R. N. et al. Interactive effects of wildfire and climate on permafrost degradation in Alaskan lowland forests. J. Geophys. Res. G Biogeosciences 120, 1619–1637 (2015).

Jafarov, E.E. et al. The effects of fire on the thermal stability of permafrost in lowland and upland black spruce forests of interior Alaska in a changing climate. Environ. Res. Lett. 8, 035030 (2013).

Reviewer #2 (Remarks to the Author):

Authors coupled field and remote sensing observations with existing soil and fire databases to study the impact of wildfires on thermokarst bog expansion rate in the Canadian peatlands. Authors report that assessments of permafrost vulnerability to climate change are essentially underestimates, as these assessments do not consider the impact of wildfires on thermokarst bog

expansion. I found this manuscript well written and conclusions interesting, and agree with author’s overall findings. I think the findings of this study should be published.

However, I have two major concerns in this study:

1) I think representing 431,000 km2 land area with six locations and 16 study sites is a over stretch. I didn’t find any scientific reasoning on selecting these sites in the methodology section. Are these sites adequately representing the environmental heterogeneity of the entire study area? I will like to see authors mention this as the limitation of the study.

2) I will like to see a separate uncertainty propagation section in this manuscript that states clearly, how the uncertainties that exists in field observations, remote sensing data, soil and fire databases has been propagated towards the final estimates of thermokarst bog expansion rates. Would the conclusions be different has these uncertainties been accounted for?

Reviewer #3 (Remarks to the Author):

(Q1) What are the major claims of the paper?

By examining a permafrost peatland chronosequence in northwestern Canada, the authors argue that fires in these landscapes increase talik formation and lateral thaw, most noticeably during a 10-30-yr period following fire. Such changes are likely to increase with climate-driven changes in fire frequency/extent, which, in turn, could impact the C balance of these ecosystems.

(Q2) Are they novel and will they be of interest to others in the community and the wider field?

The various links among climate, fire, permafrost thaw, and C balance have all been explored previously in different high-latitude systems. What is unique about this present study is the amount of information about the thermal regime, fire area, thaw rates and post-thaw accumulation histories and the integration of all of these factors in a modern peatland landscape. It's an important dataset. This study would be of wide interest to scientists studying peatlands, high-latitude systems, wildfire, climate, soil C dynamics, and biospheric feedbacks on climate. The new dataset indicates how pervasive and long-lasting (decades) the potential thermal impacts of fire can be.

(Q3) If the conclusions are not original, it would be helpful if you could provide relevant references. Is the work convincing, and if not, what further evidence would be required to strengthen the conclusions?

In general, the results are presented convincingly and effectively, but there are a few areas where additional consideration could strengthen the manuscript (discussed in more detail in the specific comments below):

(1) Remotely sensing thaw rate

Methodologically, using remote sensing to assess lateral thaw is difficult because the resolution of the imagery is often too low and the time duration between images is too short. This approach has typically only been successful (and most often with aerial photos rather than satellites) in these more southerly regions, and especially where thaw is catastrophic (what Zoltai called "top down" collapse of plateaus) rather than the slower lateral collapse of thermokarst margins. Fortunately, the combination here of (a) fire and (b) a study region located in warmer parts of the discontinuous permafrost zone may make this approach possible. Nevertheless, it would be good or the authors to include more information about the resolution of their imagery and possible error

that could propagate in the thaw coverage estimates. Fig S8 helps a bit here.

As alluded to in point 2 below, since thaw rate can decline with decreasing temperatures to the north, it becomes increasingly hard to detect with change analysis given the relatively short duration and crude spatial resolution of remotely sensed data.

(2) Geographic generalization of results

This region lies along the southern edge of permafrost, where mean annual temps (MAT) and permafrost temperatures are on the warm end of the spectrum. It's a region that is likely to already be close to the brink of thaw, and fire may be one of several mechanisms that could tip the system to a net thaw regime. Extrapolating the approach/results from this study to other regions farther north in the boreal and into the tundra (lines 326-334) therefore warrants two cautions:

(a) it may not be possible to use remote sensing in colder regions to detect lateral thaw using a similar approach.

(b) it is not clear that fires lead to thaw in a predictable/statistically known fashion in colder areas. Burned areas sometimes do not thaw in subarctic and arctic peatland landscapes. Without further work to document the probability of thaw following fire across a broad climatic gradient (beyond the scope of this study), the fire-thaw relationship remains poorly known.

Both of these caveats will make it challenging to observe similar changes elsewhere in the modern pan-boreal/arctic landscape, and changes may only transpire over the coming century as areas that are now colder shift to warmer climatic regimes similar to this region. Therefore, the greatest impact of this manuscript/approach seems to be the following two points:

(a) What is being observed in this study region might appropriately serve as an analog for what could happen as the 0 to -1 deg C MAT isotherm moves north.

(b) It could prompt greater attention from the permafrost peatland fire community to evaluate the statistical relationship between fire and thaw across a wide (0 to -8 deg C climatic gradient) to help fill in these knowledge gaps.

(3) Climate-thaw links

The proposed link between climate and permafrost thaw is not yet fully convincing. Fig. 5A purportedly shows thaw rate across a climate gradient, but further inspection of the climate data (Table S1) indicates several data gaps, so it's not clear these four sites span a climatic gradient (see specific comments below).

(4) Thaw coverage-time since fire links

It could be that I am misinterpreting the purpose of the analysis from lines 193-281, but I am curious about the use of just the 20-30-year-old landscapes here. One of the main conclusions is that the young thaw bogs show greater coverage in burned (8.6%) than in unburned (5.3%) parts of the landscape. This result then serves as the basis for the conclusion that thaw rate may increase with fire.

Although the thermal data in Fig 1 are convincing that these younger (10-30-yr post fire) time frames are a point of significant warming/thaw/talik formation, it's not clear from this approach that the 20-30 yr-old landscapes are unique in having more young thermokarst bogs in burned than in unburned areas. What about 40-50, 60+ yr-old parts of the landscape? What if they also showed this same relative difference in young thermokarst bog coverage? This could indicate that

(a) thaw is happening for reasons other than fire;
(b) fire is not always causing thaw;
(c) lateral thaw rates are temporally disconnected from peak impacts on thermal regime (10-20 yr).

What seems missing from the analysis is a demonstration that the 8.6% vs 5.3% difference is at its peak in the 20-30-yr-old landscapes relative to other sites of different post-fire age classes.

(5) Additional sources

There are instances where additional citations could be helpful:

-links between fire and C (Grosse et al. 2011)
-climate and lateral permafrost peatland thaw (Camill 2005)
-fire consumption of surface peats (various Benscoter manuscripts from Alberta)
-links between thermokarst-C accumulation/CH4 in boreal permafrost peatlands: lots of relevant/citable work has been done here

(Q4) On a more subjective note, do you feel that the paper will influence thinking in the field?

Yes, this topic is of primary importance to scientists studying high-latitude systems. The prior work from this team is also first-rate.

(Q5) Please feel free to raise any further questions and concerns about the paper.

Specific comments

Lines 112-123: This first paragraph reads more like methods statements rather than results. The emphasis on supplemental tables, figures, and peat plateau cross sections (Fig. 2) is a bit distracting and makes it difficult to key in right away on main results. Figure 3 should be the center of attention. The text starting at line 135 accomplishes this better. Maybe move lines 112-123 to the methods?

Table S2: For the site "Sixtieth" at the bottom, please flip the burned and unburned rows to be consistent with the previous three sites in this table.

Line 157: reduces

Line 166, 171: It would be helpful to cite figures after these sentences. What is the measure of fire severity used here (note: addressed later on in methods)? The reader has little insight as to how the conclusion of a lack of influence of fire severity is being reached. There may be near-complete tree mortality but fire severity can play out in other important ways that have direct impact on the peat thermal regime--especially the consumption of the peat layer by fire. Were there sites where the surface peat was only scorched vs consumed to depth? This can be difficult to assess years-to-decades post fire.

Table S1: It might be helpful to include a note at the bottom of this table that explains what the drought code is.

Lines 177-179: There is little evidence presented to substantiate this claim. The full spectrum of peat consumption (from none to 10s of cm) following wildfires on peat plateaus can be commonly observed. Peat plateau fires are commonly patchy at a local scale, often burning around wetter hummocks and consuming drier feathernoss peat. See the work in Alberta by Benscoter et al.

Fig 3, Line 188: The observations of changes across time is based on the assumption of a space for time substitution. Is this reasonable in this landscape? Are there other factors that might be confounding talik coverage, active layer depth, max soil temp, or lichen coverage with time? This can be a challenge since there do not appear to be replicate burn sites of comparable age (the plots in Figs 3a-b have no associated error bars) that can assist with assessing how variable these trends might be.

Line 200-226: This section also feels like methods. Going back and forth between methods and results can be distracting. The results start at line 227.

Line 203: Although landscapes that are 20-30 years old may be showing greatest thaw, why limit the remote sensing analysis to landscapes with just these age classes? If the analysis were performed across all post-fire peatland ages, then the authors could test statistically whether the coverage estimates in line 228 are different with burn age. It could be, for instance, that the coverage percentages of 8.6% (burned) and 5.3% (unburned) are the same in all peatland landscapes (for reasons that include but are not limited to fire, such as climate--line 234) regardless of the time since fire (to play devil's advocate, for instance, one could pose an alternate hypothesis that ought to be tested: 8.6%/5.3% are the burned/unburned coverages of young thermokarst regardless of time since fire). If that were the case, the fire impacts on lateral thaw would have to be reinterpreted with respect to the thermal data in Figs 3A-B.

Lines 202-205: As suggested in the previous statement, rather than assuming this a priori, let the statistical analysis across landscapes of all post-fire ages demonstrate this point. Otherwise, the coverage estimates (line 228) are supporting a pre-determined conclusion that may not be correct.

Lines 229-30: And this variability is of concern for the reasons stated in the previous two points--young thermokarst may or may not be present in landscapes regardless of fire. In Fig. 5A, for instance, there appears to be more young thermokarst % in the unburned landscape at the Sixtieth site compared to the burned landscape in either the Trout Lake or Zama sites.

Line 234: The relationship between climate and thermokarst development (from a MAT range of about -1 to -5 deg C) has been reported previously in Manitoba (see Camill 2005 Climatic Change).

Lines 231-234: It's not clear from Fig 1 or 5A how these sites relate to climate. The authors state in the Fig. 5A caption that the sites are ordered from left to right according to decreasing MAT, but mean air temps are not reported in Table S1 for two of the four sites (Trout Lake or Sixtieth). The MATs reported in Table S1 (burned MAT/unburned MAT) are as follows:

Sixtieth = NA/NA
FT Simp = -0.15/-0.81
Trout = NA/NA
Zama = NA, -1.57

Is it possible to fill these gaps to better support the climate-thermokarst relationship?

Another point: If the temperature range is only from 0 to -1.57 deg C, the authors need to consider that this is a relatively narrow temperature range on the warm end of the climate/permafrost spectrum for interpreting climate-thermokarst dynamics. If the authors were to push northwards into regions where MAT was below -2, -5, -7 deg C, for instance, they may observe less thermokarst even with burning. This is where a climate x fire interaction would likely show up. While the results in this study are interesting and useful, broader generalizations between climate, fire, and thermokarst may not be warranted--or done so with caution since this study only focuses on one narrow climatic range for the warm end of the discontinuous permafrost

zone.

Fig 5. The panels need to be labeled A and B.

Lines 236-238: This statement may help, but I'm not sure as to what landscapes we're talking about--is it the four peatland sites or all of them?

Lines 256-272: This is an interesting analysis that, in part, addresses some of the concerns raised previously, and the assumptions made are necessary (although see next point below), but as mentioned earlier, why not let the remote sensing data across all burn age classes inform this analysis?

Lines 263-264: The rate of transition of young to mature thermokarst bogs is dependent on the local hydrology, peat accumulation rates, and bulk density increases in the thermokarst bogs. These can lead to variable transitions and ought to be mentioned as caveats in the methods.

Lines 292, 297-299: Be cautions of overinterpretation. "Clearly" in line 292 is an overstatement. Without the analysis of thaw % by fire age across all landscape fire age classes, the authors need to be more cautious with these interpretations. Line 297 needs to be restated. Peat plateaus can burn but not thaw, especially in colder regions.

Lines 317-323: The citations for CH_4, CO_2, and peat accumulation changes with thermokarst could be improved. There is a large body of research on each of these.

Lines 326-334: As alluded to above, the authors need to be cautions when generalizing beyond the southern edge of the sporadic discontinuous permafrost zone. Permafrost in colder regions will likely not respond, at least currently/initially, as that in this study. While it is tempting to extrapolate these results to other boreal areas and the tundra, that would almost certainly overestimate the effect of fire on thaw. Although beyond the scope of this manuscript, what is needed, instead, is a probabilistic framework that describes the likelihood of thaw given a burn across a broad MAT gradient (0 to -8 deg C). Until this kind of analysis is performed, the peatland scientific community needs to be cautions in extrapolating dynamics from the southern boundary across the pan-boreal/arctic regions.

Line 383: 3-m

Line 425: What is the resolution of the remotely sensed data? It needs to be fairly high to be able to resolve decadal-scale changes in thermokarst.

Response to reviewer comments on "Wildfire as a major driver of recent permafrost thaw in boreal peatlands." by Gibson et al.

We would like to thank the reviewers and editors for their constructive comments on our manuscript. We believe that we have addressed all reviewer comments and that the manuscript has been improved as a result of the revision process. Improvements have been made to communicate limitations to the interpretation of the study, but we note that the revisions have not substantially changed our results or conclusions qualitatively or quantitatively. Below we address specific revisions in response to each reviewer comment. Line number references refer to those in the revised manuscript.

Reviewers' comments:

<u>Reviewer #1</u>

This study addresses an important and timely question regarding the growing role of wildfire in influencing permafrost thaw in peatlands and the expansion of thermokarst bogs. Understanding and quantifying the effects of wildfire on permafrost thaw is essential for predicting future greenhouse gas emissions and feedbacks to global climate change, and has not yet been well-documented. This paper will be of great interest to permafrost scientists, ecologists, climate scientists, and the broader community interested in global change. The authors clearly demonstrate the temporal trajectory of permafrost thaw and recovery in peat plateaus after wildfire through field sampling, and use remote sensing to document and scale up the increased rates of thermokarst bog expansion after fire. The study is well-designed and the presentation is polished. I have only a few minor comments for the authors to consider.

line 14: Perhaps specify that "effects of wildfire *in peat plateaus* were found to last for 30 years…". As currently written, the sentence appears to suggest that effects of fire on permafrost overall only last for 30 years, contrary to what is stated in the discussion, that the effects of fire on permafrost via thermokarst bog expansion is considered irreversible at relevant timescales.

Our response: We agree that this is an important point to highlight. The sentence in the abstract has been revised to read (L14-16): "Effects of wildfire on permafrost peatlands last for 30 years and include a warmer and deeper active layer, and spatial expansion of continuously thawed soil layers (taliks)." We avoid using the term peat plateau in the abstract (instead here we just say permafrost peatlands) as it may need a definition/description, which we have in the introduction.

figure 3/lines 149-161: Interesting how clear and consistent these changes in permafrost and vegetation are over time since fire. This timeline of degradation/recovery is consistent with the theoretical work presented in Jafarov et al 2013. Their thermal model simulations showed permafrost recovery in Alaskan peatlands approx. 30 years after fire as well, and also found only minor impacts of fire severity in deep organic soils.

Our response: Yes, our results are consistent with Jafarov et al.'s modelling results. We have now included a reference to this article (L269).

lines 187-188: Do you have any information regarding the depth of the taliks? It'd be interesting to know how deeply a talik could form and allow subsequent permafrost recovery in the current climatic conditions.

Our response: Our frost probe was 150 cm, so we could not tell the position of the base of the thawed taliks when it was any deeper. Recent work by Connan et al. (2018) tracked active layer depths in peat plateaus in the discontinuous permafrost zone of the Northwest Territories and found that the base of taliks can be >180 cm in summer. Pushing a frost probe deeper than 150-180 cm is difficult. From experience in sampling soil cores from peat plateaus, we have found that most of the visible excess ice is found as ice lenses closer to the base of the peat profile. Thus surface collapse doesn't seem really happen unless thaw reaches layers near the peat base. With most of our sites having 3-5 m of peat, we estimate that taliks on top of peat plateaus could be quite deep (2-3 m) without causing irreversible permafrost thaw. As shown in a photo below – if surface collapse does happen, and a depression with wetter conditions establish, then it seems very difficult for this process to not develop into new thermokarst bogs – at least in this region with relatively warm climates for being a permafrost region.

lines 303-305: You might need to explain somewhere in the discussion why the permafrost thaw in peat plateau centers is reversible but the permafrost thaw through thermokarst bog development at plateau edges is not reversible (the role of water and vegetation in soil thermal regimes?). Also, I'm curious if you found that the thawing of permafrost in plateau centers after fire resulted in the initiation of any small thermokarst depressions that subsequently stabilized. Brown et al 2015 noted that ice-rich permafrost thawed deeply in an Alaskan peatland after an old fire, but eventually refroze, leaving behind thermokarst microtopography.

Our response: The discussion on the reversibility and non-reversibility of permafrost thaw within peat plateaus and at the edges of peat plateaus has been expanded (Line 272 – 277): "Permafrost thaw within peat plateaus (i.e. active layer deepening) appeared reversible under the recent climate since pre-fire soil thermal conditions were able to recover fully after 30 years as vegetation recovered to pre-fire conditions. However, permafrost thaw that at the edges of peat plateaus (i.e. thermokarst bog development) is considered irreversible given that these ecosystems undergo a complete successional shift to a new vegetation community that also includes strongly altered soil thermal and hydrological regimes."

We did observe some small thermokarst bogs within burned peat plateaus which we believe formed after the fire, as in the photo below. The dead trees in this thermokarst are likely trees that started regenerating after the fire, and then the thermokarst development caused surface collapse and inundation. Once the thermokarst bogs have developed to the point seen below, with standing water and Sphagnum colonization, it is extremely unlikely under current climate that there will be permafrost recovery given the greater thermal conductivity of these wet surfaces.

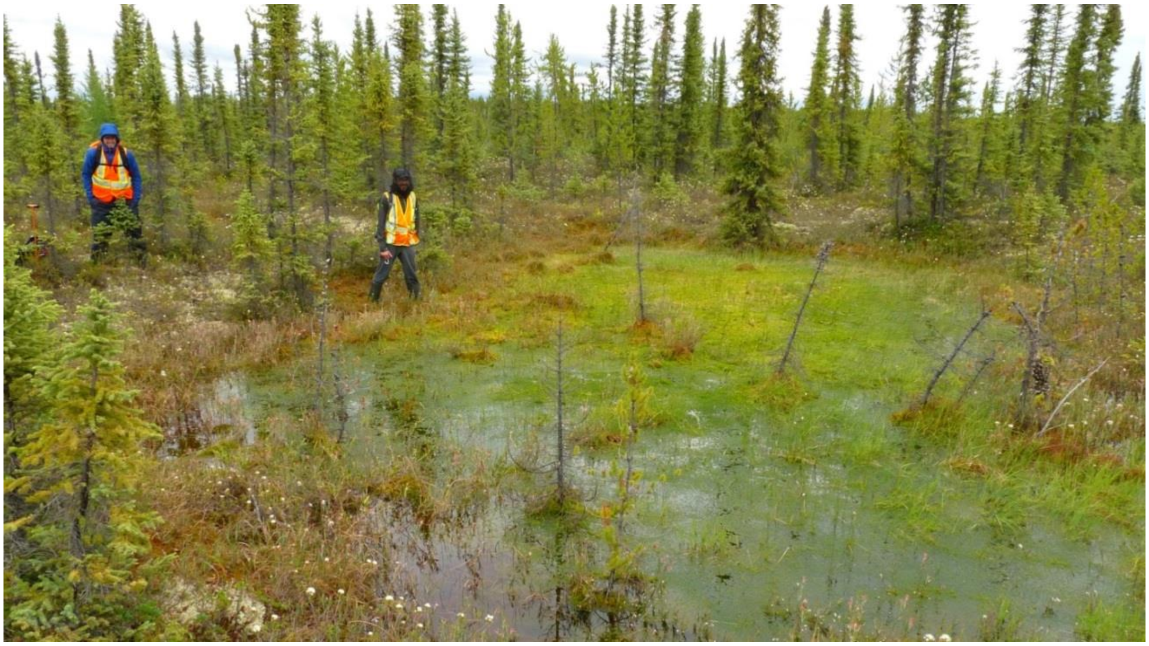

References

Brown, D. R. N. et al. Interactive effects of wildfire and climate on permafrost degradation in Alaskan lowland forests. J. Geophys. Res. G Biogeosciences 120, 1619–1637 (2015).

Jafarov, E.E. et al. The effects of fire on the thermal stability of permafrost in lowland and upland black spruce forests of interior Alaska in a changing climate. Environ. Res. Lett. 8, 035030 (2013).

Reviewer #2 (Remarks to the Author):

Authors coupled field and remote sensing observations with existing soil and fire databases to study the impact of wildfires on thermokarst bog expansion rate in the Canadian peatlands. Authors report that assessments of permafrost vulnerability to climate change are essentially underestimates, as these assessments do not consider the impact of wildfires on thermokarst bog expansion. I found this manuscript well written and conclusions interesting, and agree with author's overall findings. I think the findings of this study should be published.

However, I have two major concerns in this study:

1) I think representing 431,000 km2 land area with six locations and 16 study sites is a over stretch. I didn't find any scientific reasoning on selecting these sites in the methodology section. Are these sites adequately representing the environmental heterogeneity of the entire study area? I will like to see authors mention this as the limitation of the study.

Our response: Overall, we do believe that our sites are representative of the chosen study region. Peat plateaus have little variation in terms of vegetation communities, structure, and ground microtopography within the study region. Thick peat accumulation and permafrost conditions are strong controls on site hydrology and nutrient availability, and thus ensures the similarity between peat plateau sites. In short, peat plateaus are

probably one of the most suitable ecosystems for conducting space-for-time chronosequence studies. Furthermore, while all our study locations were found in the southern part of the identified study region, some of these locations (e.g. the Zama and Trout lake locations) were located at higher elevation (~300 to 400 m higher than elevations in the northern part of the study region). Elevation is a strong control on the local climate and thus each of the three Level III ecoregions found within the study region (mid-boreal, high boreal, and low subarctic) were represented by at least one study location. We have included a sentence in the methods to emphasize this (L333-337):

"While all locations were found in the southern part of the identified study region, each of the three level III ecoregions of within the study region (mid-boreal, high boreal, and low subarctic ecoregions) were represented by at least one study location, with the colder low subarctic climate ecoregion represented in southern part of the study region by a location at higher elevation (Zama)."

We have, however, made several changes to emphasize that the magnitude and duration of effects of wildfire on peatland permafrost stability found in this study should not be directly extrapolated to other regions, given differences in climate, peatland development history, and fire regimes:

L12-14: "In this study of western Canadian permafrost peatlands, we assess impacts of wildfire on soil thermal regime and rate of thermokarst bog expansion resulting from complete permafrost thaw."

L265-268: "In this study, we showed that wildfire in boreal peatlands within the discontinuous permafrost zone cause permafrost thaw through active layer deepening and talik expansion on peat plateaus, but also through accelerated thermokarst bog development along peat plateau edges."

L301-304: "While the magnitude of effects from wildfire are likely to differ depending on the regional climate, peat plateaus under similar climates and fire regimes as described in this study are widespread also in boreal Alaska and in the Hudson Bay lowlands."

The criteria for site selection is described in the methods (L324-333): "All peat plateau sites were identified using satellite imagery available in Google Earth in combination with burn area polygons from the Canadian National Fire Database, which includes information on the size and date of the fires that had affected the chosen burned sites (Supplementary Table 1) 53. Locations were chosen to include burned sites that burned between 2 to 49 years prior to the study in 2016, but locations were also required to have nearby unburned sites and for all sites to be accessible either by foot from nearby roads or by short helicopter trips from Fort Simpson."

With these selection criteria, we believe that we included peat plateau sites within <u>all</u> reasonably accessible burn-area polygons located between High Level, AB, and Wrigley, NT. Hence, there is no bias towards large or small fires, or towards large or small peatlands, for example.

2) I will like to see a separate uncertainty propagation section in this manuscript that states clearly, how the uncertainties that exists in field observations, remote sensing data, soil and fire databases has been propagated towards the final estimates of thermokarst bog expansion

rates. Would the conclusions be different has these uncertainties been accounted for?

Our response: We have made modification to the paragraph in the methods section which describes the methodology for estimating area of thermokarst bog development within the study region L462-490.

We now explicitly state that we consider the error of the soils map and the burn-area polygons to be negligible (L467-468): "For the scaling, we assumed that the soils maps and fire maps have negligible errors."

We acknowledge that there likely is significant uncertainty in these spatial data sources, but we have no way to assume neither any bias nor the uncertainty of them.

We also explicitly state that we assume that the full cumulative effect of wildfire on thermokarst bog development happens during the first 30 years after fire (L468-470): "We also assumed that the accelerated rate of thermokarst bog development lasted for 30 years after fire, i.e. that our analysis of peatlands that burned 20-30 years ago accurately captured the cumulative impact of wildfire on thermokarst bogs development."

Our remote sensing analysis does not rule out the potential for fire to continue to influence the rate of thermokarst bog development beyond 30 years, but given the recovery of the soil thermal regime we believe this effect to be marginal at most. This limitation of the remote sensing analysis is now also explicitly stated (L251-253): "Our remote sensing analysis can not rule out any effects beyond 30 years after fire, and as such this is potentially a conservative measure of the effect of wildfire on thermokarst bog development."

The only other factor used to estimate the area of thermokarst bog development within the study region is the rate of thermokarst development estimated at the four paired sites included in the remote sensing analysis. The 95% confidence intervals of these rates are based on 1.96 SD among rates from the four individual sites. Thus we assume that we have captured the variability within the study region using these four sites. We have addressed the representativeness of the included study sites for the study region in the comment above. The methodology for estimating thermokarst bog expansion rate and its uncertainty at each individual site is described in the methods, L434-460.

<u>Reviewer #3 (Remarks to the Author):</u>

(Q1) What are the major claims of the paper?

By examining a permafrost peatland chronosequence in northwestern Canada, the authors argue that fires in these landscapes increase talik formation and lateral thaw, most noticeably during a 10-30-yr period following fire. Such changes are likely to increase with climate-driven changes in fire frequency/extent, which, in turn, could impact the C balance of these ecosystems.

(Q2) Are they novel and will they be of interest to others in the community and the wider field?

The various links among climate, fire, permafrost thaw, and C balance have all been explored previously in different high-latitude systems. What is unique about this present study is the amount of information about the thermal regime, fire area, thaw rates and post-thaw accumulation histories and the integration of all of these factors in a modern peatland landscape. It's an important dataset. This study would be of wide interest to scientists studying peatlands, high-latitude systems, wildfire, climate, soil C dynamics, and biospheric feedbacks on climate. The new dataset indicates how pervasive and long-lasting (decades) the potential thermal impacts of fire can be.

(Q3) If the conclusions are not original, it would be helpful if you could provide relevant references. Is the work convincing, and if not, what further evidence would be required to strengthen the conclusions?

In general, the results are presented convincingly and effectively, but there are a few areas where additional consideration could strengthen the manuscript (discussed in more detail in the specific comments below):

(1) Remotely sensing thaw rate

Methodologically, using remote sensing to assess lateral thaw is difficult because the resolution of the imagery is often too low and the time duration between images is too short. This approach has typically only been successful (and most often with aerial photos rather than satellites) in these more southerly regions, and especially where thaw is catastrophic (what Zoltai called "top down" collapse of plateaus) rather than the slower lateral collapse of thermokarst margins. Fortunately, the combination here of (a) fire and (b) a study region located in warmer parts of the discontinuous permafrost zone may make this approach possible. Nevertheless, it would be good or the authors to include more information about the resolution of their imagery and possible error that could propagate in the thaw coverage estimates. Fig S8 helps a bit here.

As alluded to in point 2 below, since thaw rate can decline with decreasing temperatures to the north, it becomes increasingly hard to detect with change analysis given the relatively short duration and crude spatial resolution of remotely sensed data.

Our response: The satellite image spatial resolution (WorldView2) is 0.6 m, which is stated in the methods section (L407), and in figure 4 legend (L693). This fine resolution is required to adequately assess young thermokarst bog extents, since these features often are < 10 m wide. As far as I'm aware, it is the highest resolution image quality that can be commercially acquired for the region.

As shown in Supplementary Figure 8, we did not find a bias in the satellite image classification of young thermokarst bog extents when compared to our field assessments. As such, we assume absence of bias in our young thermokarst bog estimates when scaling our results to regional thaw coverages. This is now explicitly stated in the results section, L193-198: "The distance between field-determined ecological transitions and transitions in the supervised classification was within 1 m 80% of the time, and without bias, in both burned and unburned sections (Supplementary Fig. 8). The field validation thus showed that the supervised classification of young thermokarst bogs would be able to provide a both precise and unbiased measure of differences in thermokarst bog expansion between burned and unburned peatland parts."

The description of the error propagation for the scaling of thaw areas has been expanded, and now explicitly states all assumptions, L462-490 – for details see answer to comment 2 from reviewer 2 above.

(2) Geographic generalization of results

This region lies along the southern edge of permafrost, where mean annual temps (MAT) and permafrost temperatures are on the warm end of the spectrum. It's a region that is likely to already be close to the brink of thaw, and fire may be one of several mechanisms that could tip the system to a net thaw regime. Extrapolating the approach/results from this study to other regions farther north in the boreal and into the tundra (lines 326-334) therefore warrants two cautions:

(a) it may not be possible to use remote sensing in colder regions to detect lateral thaw using a similar approach.

(b) it is not clear that fires lead to thaw in a predictable/statistically known fashion in colder areas. Burned areas sometimes do not thaw in subarctic and arctic peatland landscapes. Without further work to document the probability of thaw following fire across a broad climatic gradient (beyond the scope of this study), the fire-thaw relationship remains poorly known.

Both of these caveats will make it challenging to observe similar changes elsewhere in the modern pan-boreal/arctic landscape, and changes may only transpire over the coming century as areas that are now colder shift to warmer climatic regimes similar to this region. Therefore, the greatest impact of this manuscript/approach seems to be the following two points:

(a) What is being observed in this study region might appropriately serve as an analog for what could happen as the 0 to -1 deg C MAT isotherm moves north.

(b) It could prompt greater attention from the permafrost peatland fire community to evaluate the statistical relationship between fire and thaw across a wide (0 to -8 deg C climatic gradient) to help fill in these knowledge gaps.

Our answer: We note that the study included sites that span a climate gradient representative of the study region. While all sites are located in the southern part of the study region, some of the sites are located at relatively higher elevation than found further north and thus have a climate that is characteristic of the northern parts of the region. The identified study region includes three Level III ecoregions: Low Subarctic, High Boreal and Mid Boreal (See Ecological Regions of the Northwest Territories: Taiga Plains), and all three of these regions are represented by both field sites and remote sensing sites. Our monitoring of air temperature shows that our field sites had mean annual temperatures for our year of study that ranged between -3.1 and -0.1°C, which well represents the range for the identified study region – with some exception of the very northernmost parts of the study region. However, using the transition from discontinuous to continuous permafrost was deemed the best split within the Taiga Plains to limit our study region. We now characterize the representativeness of the study sites for the study region a bit more, L101-105:

“Differences in soil thermal regimes were considered to be primarily due to differences in fire histories among sites, since sites all had similar peat depths, and current or pre-fire tree densities, and since the variability in mean annual air temperatures between -0.8 and

-3.1˚C among unburned sites did not explain any of the variability in their active layer depth or talik coverage (p>0.5, linear regressions) (Supplementary Table 1).",

L204-211:

"The Zama and Trout Lake sites were located at elevations ~300 m higher than the two other sites, and are indicated to have low subarctic climate in contrast to the lower elevation sites that are located in high boreal or mid-boreal ecoregions. Fied data also confirmed that the Zama site had a colder climate than the Fort Simpson site during the year of our study, at -0.8 and -3.1˚C. While no direct climate data for the 60th parallel site was available, this site was located both at a low elevation and in the southernmost part of the study region, and would thus be expected to have the warmest climate, explaining the greatest young thermokarst bog coverage within the unburned areas among our four sites."

and L333-337:

"While all locations were found in the southern part of the identified study region, each of the three level III ecoregions of within the study region (mid-boreal, high boreal, and low subarctic ecoregions) were represented by at least one study location, with the colder low subarctic climate ecoregion represented in southern part of the study region by a location at higher elevation (Zama)."

We have also made changes to caution the extrapolation of our results to regions further north, L301-304:

"While the magnitude of effects from wildfire are likely to differ depending on the regional climate, peat plateaus under similar climates and fire regimes as described in this study are widespread also in boreal Alaska and in the Hudson Bay lowlands."

(3) Climate-thaw links

The proposed link between climate and permafrost thaw is not yet fully convincing. Fig. 5A purportedly shows thaw rate across a climate gradient, but further inspection of the climate data (Table S1) indicates several data gaps, so it's not clear these four sites span a climatic gradient (see specific comments below).

Our answer: Table S1 indicates the mean annual temperature at the field sites, not the remote sensing sites. The Zama site is the only site where we had field and remote sensing analysis done for the very same location. The fort Simpson field site and remote sensing sites were ~25 km apart. No field site was close to the Trout Lake or 60th remote sensing sites.

We thus do not have direct measured air temperatures from all the four remote sensing sites. However, the Zama site with the lowest coverage of young thermokarst bogs was also our coldest field site (-3.1˚), and it was also the site located at the highest elevation, and the only site located within a low subarctic ecoregion. Trout Lake site is located at similar elevation as Zama, and is thus likely to have a cooler climate than the lower elevation FtSimpson and 60th parallel sites. The field site at FtSimpson accordingly had a mean annual temperature of -0.8˚C. Given the lack of direct data to support our statement of differences in climate, we have now cautioned our interpretation of the differences

between remote sensing sites, but still speculate that likely differences between sites are driven by differences in climate, and this is indicated in the figure legend L702-703:

"Sites are ordered left to right by likely decreasing mean annual air temperature, see text for justification."

And this justification is found in the results section, L201-211:

"There was, however, a large variability in young thermokarst bog coverage among unburned sites, between 2.7 and 10.5%, with the lowest coverage at the higher elevation sites, Zama and Trout Lake (Supplementary Table 2). The Zama and Trout Lake sites were located at elevations ~300 m higher than the two other sites, and are indicated to have low subarctic climate in contrast to the lower elevation sites that are located in high boreal or mid-boreal ecoregions. Fied data also confirmed that the Zama site had a colder climate than the Fort Simpson site during the year of our study, at -0.8 and -3.1˚C. While no direct climate data for the 60th parallel site was available, this site was located both at a low elevation and in the southernmost part of the study region, and would thus be expected to have the warmest climate, explaining the greatest young thermokarst bog coverage within the unburned areas among our four sites."

(4) Thaw coverage-time since fire links

It could be that I am misinterpreting the purpose of the analysis from lines 193-281, but I am curious about the use of just the 20-30-year-old landscapes here. One of the main conclusions is that the young thaw bogs show greater coverage in burned (8.6%) than in unburned (5.3%) parts of the landscape. This result then serves as the basis for the conclusion that thaw rate may increase with fire.

Although the thermal data in Fig 1 are convincing that these younger (10-30-yr post fire) time frames are a point of significant warming/thaw/talik formation, it's not clear from this approach that the 20-30 yr-old landscapes are unique in having more young thermokarst bogs in burned than in unburned areas. What about 40-50, 60+ yr-old parts of the landscape? What if they also showed this same relative difference in young thermokarst bog coverage? This could indicate that

(a) thaw is happening for reasons other than fire;
(b) fire is not always causing thaw;
(c) lateral thaw rates are temporally disconnected from peak impacts on thermal regime (10-20 yr).

What seems missing from the analysis is a demonstration that the 8.6% vs 5.3% difference is at its peak in the 20-30-yr-old landscapes relative to other sites of different post-fire age classes.

Our response: The difference in young thermokarst bog coverage between areas, within the same pair, we consider the cumulative impact of fire up until the date of the satellite image acquisition. Based on the field data, we do not expect that thermokarst bog expansion rates are greater in burned than unburned areas after >30 years, but this is an assumption that our data cannot ascertain. This limitation is now highlighted, L176-183:

"The four peatlands were chosen for this analysis since the 20 to 30 years since fire coincided with the duration over which wildfire was found to influence peat plateau soil

thermal regime, and thus likely also the period over which it would influence the rate of thermokarst bog development. Hence, we expected the majority of the cumulative effect of wildfire on thermokarst bog development to be accounted for by choosing sites that burned 20-30 year ago. However, analysis using the chosen sites can not rule out effects of wildfire on thermokarst bog expansion extending beyond this time frame, and as such our analysis is potentially conservative."

We further agree that it would have been interesting to include sites that burned both more recently (e.g. 5 to 10 years after fire) and later (40-50 years after fire). However, we were limited in terms of funding and time, and were thus limited to carrying out this analysis at 4 paired sites. As such, we decided that the best approach was to assess the variability among sites that burned 20-30 years ago, which likely represented the majority of the cumulative effect of wildfire.

Our results showed that despite differences among sites in terms of young thermokarst bog extents in the unburned sites, the difference in young thermokarst extents between burned and unburned areas at the same site was consistent among the four sites. This was confirmed with the two-way ANOVA. We consider this strong evidence that fire was the cause of the difference between young thermokarst bog extents between burned and unburned areas within each pair.

(5) Additional sources

There are instances where additional citations could be helpful:

-links between fire and C (Grosse et al. 2011)
-climate and lateral permafrost peatland thaw (Camill 2005)

Our answer: These references have been incorporated in our revisions.

-fire consumption of surface peats (various Benscoter manuscripts from Alberta)

Our response: We have instead included a reference on fire consumption of surface peat that was done within the study region – the Benscoter paper is from outside the permafrost region and non-permafrost bog combustion appears different from peat plateau peat combustion.

Walker, X. J., Baltzer, J. L., Cumming, S. G., Day, N. J., Johnstone, J. F., Rogers, B. M., … Mack, M. C. (2018). Soil organic layer combustion in boreal black spruce and jack pine stands of the Northwest Territories, Canada. *International Journal of Wildland Fire*, *27*, 125–134. https://doi.org/10.1071/WF17095

-links between thermokarst-C accumulation/CH4 in boreal permafrost peatlands: lots of relevant/citable work has been done here

Our response: We have added a few key references:

Jones, M. C., Harden, J., O'Donnell, J., Manies, K., Jorgenson, T., Treat, C., & Ewing, S. (2017). Rapid carbon loss and slow recovery following permafrost thaw in boreal peatlands. *GLOBAL CHANGE BIOLOGY*, *23*(3), 1109–1127. https://doi.org/10.1111/gcb.13403

O'Donnell, J. A., Jorgenson, M. T., Harden, J. W., McGuire, A. D., Kanevskiy, M. Z., & Wickland, K. P. (2012). The Effects of Permafrost Thaw on Soil Hydrologic, Thermal, and Carbon Dynamics in an Alaskan Peatland. *ECOSYSTEMS*, *15*(2), 213–229. https://doi.org/10.1007/s10021-011-9504-0

Schuur, E. A. G., McGuire, A. D., Schadel, C., Grosse, G., Harden, J. W., Hayes, D. ., … Vonk, J. E. (2015). Climate change and the permafrost carbon feedback. *Nature*, *250*, 217–227. https://doi.org/10.1038/nature14338

(Q4) On a more subjective note, do you feel that the paper will influence thinking in the field?

Yes, this topic is of primary importance to scientists studying high-latitude systems. The prior work from this team is also first-rate.

(Q5) Please feel free to raise any further questions and concerns about the paper.

Specific comments

Lines 112-123: This first paragraph reads more like methods statements rather than results. The emphasis on supplemental tables, figures, and peat plateau cross sections (Fig. 2) is a bit distracting and makes it difficult to key in right away on main results. Figure 3 should be the center of attention. The text starting at line 135 accomplishes this better. Maybe move lines 112-123 to the methods?

Our response: We have kept the paragraph in the results section. The reason is that we consider it necessary to describe how we used the thaw-depth data to define and estimate active layer depth and talik coverage. It is our assessment that this is necessary to understand and interpret Figure 3, which contains our main results for the field data. If the format of the journal was to have the methods section between the introduction and the results, we would agree that this paragraph would be in the methods section. We are open to moving the paragraph into the methods section if recommended by the editor.

Table S2: For the site "Sixtieth" at the bottom, please flip the burned and unburned rows to be consistent with the previous three sites in this table.

Our response: This has been corrected in our revisions.

Line 157: reduces

Our response: This has been corrected in our revisions.

Line 166, 171: It would be helpful to cite figures after these sentences. What is the measure of fire severity used here (note: addressed later on in methods)? The reader has little insight as to

how the conclusion of a lack of influence of fire severity is being reached. There may be near-complete tree mortality but fire severity can play out in other important ways that have direct impact on the peat thermal regime--especially the consumption of the peat layer by fire. Were there sites where the surface peat was only scorched vs consumed to depth? This can be difficult to assess years-to-decades post fire.

Our response: This paragraph has been modified, L137-143:

"Fire severity did not appear to have any influence on the post-fire soil thermal regime. Fire severity is generally higher during droughts and for fires that occur later in the season[35]. However, neither the Canadian drought code, which is a rating of the average moisture content of deep organic layers, nor the Julian date of the fires explained any of the variability in soil thermal regime among the burned peat plateau sites ($p>0.5$, multiple linear regressions with active layer depth or talik coverage as dependent variables, and years since fire, Julian date, and drought code as independent variables)."

As seen, we now quickly introduce Julian Date and Drought Code as our available indices of burn severity, and reference to Supplementary Table 1 where this data can be found for each site. We also describe how we tested whether these indices could help explain any residual variability of active layer depth or talik coverage after taking into account time since fire using multiple linear regressions. Further into the paragraph we now acknowledge that there likely was variability in depth of burn among sites and explain why we do not consider it to be as consequential as for forests/peatlands with thinner organic soils, L150-153:

"While there likely was substantial variability among sites with regards to depth of burn among our sites, the thick organic soils of the peat plateaus prevented complete peat combustion, which likely explain the consistent trajectory of vegetation recovery across burned sites (Fig. 3c)."

Table S1: It might be helpful to include a note at the bottom of this table that explains what the drought code is.

Our response: This has been incorporated in our revisions.

Lines 177-179: There is little evidence presented to substantiate this claim. The full spectrum of peat consumption (from none to 10s of cm) following wildfires on peat plateaus can be commonly observed. Peat plateau fires are commonly patchy at a local scale, often burning around wetter hummocks and consuming drier feathernoss peat. See the work in Alberta by Benscoter et al.

Our response: Our point is that even extreme fire severity, from a peat combustion perspective, is highly unlikely to cause complete loss of peat profiles that are 2-6 m as is common in the study region. In regions with generally thinner peats, combustion is much more likely to cause near-complete loss of the peat layer, L150-153:

"While there likely was substantial variability among sites with regards to depth of burn among our sites, the thick organic soils of the peat plateaus prevented complete peat combustion, which likely explain the consistent trajectory of vegetation recovery across burned sites (Fig. 3c)."

Fig 3, Line 188: The observations of changes across time is based on the assumption of a space for time substitution. Is this reasonable in this landscape? Are there other factors that might be confounding talik coverage, active layer depth, max soil temp, or lichen coverage with time? This can be a challenge since there do not appear to be replicate burn sites of comparable age (the plots in Figs 3a-b have no associated error bars) that can assist with assessing how variable these trends might be.

Our response: We do think a space-for-time substitution is largely reasonable in this landscape. Peat plateaus in the region exhibit relatively little variability in terms of vegetation composition, structure, and microtopography – which is a result of how a thick peat profile and permafrost presence strongly dictates hydrology and nutrient availability. Still, potential confounding factors could include variations in climate that are found within the study region, and possibly time since permafrost aggradation. We assume that the variability among the 6 unburned sites indicated in Figure 3 is related to such effects, as well as random effects by studying a specific plot within a peat plateau. We believe this variability is also likely to be seen among burned sites, if we had been able to include more sites. We note that the variability among unburned sites is much smaller than the variability among burned sites, strongly suggesting that it is in fact an effect of fire and that the trend with time since fire is not a random effect.

Including more sites was not possible for this study, as there were no more burned sites available by foot from roads within the study region. We have no reason to believe that the burned sites studied would have introduced a bias that led to the patterns in figure 3, and thus the most parsimonious explanation is that the general patterns are due to an effect of fire.

Line 200-226: This section also feels like methods. Going back and forth between methods and results can be distracting. The results start at line 227.

Our response: We have kept the paragraph in the results section. The reason is that we consider it necessary to describe some of the data handling and its rational in order for a reader to understand and interpret Figure 5, which contains our main results for the remote sensing data. If the format of the journal was to have the methods section between the introduction and the results, we would agree that this paragraph would be in the methods section. We are open to moving the paragraph into the methods section if recommended by the editor.

Line 203: Although landscapes that are 20-30 years old may be showing greatest thaw, why limit the remote sensing analysis to landscapes with just these age classes? If the analysis were performed across all post-fire peatland ages, then the authors could test statistically whether the coverage estimates in line 228 are different with burn age. It could be, for instance, that the coverage percentages of 8.6% (burned) and 5.3% (unburned) are the same in all peatland landscapes (for reasons that include but are not limited to fire, such as climate--line 234) regardless of the time since fire (to play devil's advocate, for instance, one could pose an alternate hypothesis that ought to be tested: 8.6%/5.3% are the burned/unburned coverages of young thermokarst regardless of time since fire). If that were the case, the fire impacts on lateral thaw would have to be reinterpreted with respect to the thermal data in Figs 3A-B.

Our response: The difference in young thermokarst bog coverage between areas, within the same pair, we consider the cumulative impact of fire up until the date of the satellite image acquisition. Based on the field data, we do not expect that thermokarst bog

expansion rates are greater in burned than unburned areas after >30 years, but this is an assumption that our data cannot ascertain. This limitation is now highlighted, L176-183:

"The four peatlands were chosen for this analysis since the 20 to 30 years since fire coincided with the duration over which wildfire was found to influence peat plateau soil thermal regime, and thus likely also the period over which it would influence the rate of thermokarst bog development. Hence, we expected the majority of the cumulative effect of wildfire on thermokarst bog development to be accounted for by choosing sites that burned 20-30 year ago. However, analysis using the chosen sites can not rule out effects of wildfire on thermokarst bog expansion extending beyond this time frame, and as such our analysis is potentially conservative."

We further agree that it would have been interesting to include sites that burned both more recently (e.g. 5 to 10 years after fire) and later (40-50 years after fire). However, we were limited in terms of funding and time, and were thus limited to carrying out this analysis at 4 paired sites. As such, we decided that the best approach was to assess the variability among sites that burned 20-30 years ago, which likely represented the majority of the cumulative effect of wildfire.

Our results showed that despite differences among sites in terms of young thermokarst bog extents in the unburned sites, the difference in young thermokarst extents between burned and unburned areas at the same site was consistent among the four sites. This was confirmed with the two-way ANOVA. We consider this strong evidence that fire was the cause of the difference between young thermokarst bog extents between burned and unburned areas within each pair.

Lines 202-205: As suggested in the previous statement, rather than assuming this a priori, let the statistical analysis across landscapes of all post-fire ages demonstrate this point. Otherwise, the coverage estimates (line 228) are supporting a pre-determined conclusion that may not be correct.

Our response: See answer just above.

Lines 229-30: And this variability is of concern for the reasons stated in the previous two points--young thermokarst may or may not be present in landscapes regardless of fire. In Fig. 5A, for instance, there appears to be more young thermokarst % in the unburned landscape at the Sixtieth site compared to the burned landscape in either the Trout Lake or Zama sites.

Our response: We do not consider this a concern for our estimate of the effect of wildfire, since our estimate of the effect of wildfire is based on the difference between burned and unburned areas within each pair. Within each pair we consider the fire history to be the main difference between the burned and unburned areas, since they are part of the same peatland complex, and thus have the same climate and likely the same development aside from the recent fire. The difference between the burned and unburned areas was consistent across the four sites, as emphasized by the two-way ANOVA, L213-219:

"Despite the apparent influence of climate on young thermokarst bog coverage, we found a consistent effect of wildfire among the four sampled peatlands, with 3.4 ±0.5% (±1 SD) greater young thermokarst coverage in burned than unburned peatland parts. Accordingly, a two-way ANOVA of young thermokarst bog coverage found strong effects of both site and fire history but no significant interactive effect (Supplementary Table 6).

As such, the relative effect of wildfire on rate of thermokarst bog expansion appeared much greater at colder sites (Fig. 5a).”

Line 234: The relationship between climate and thermokarst development (from a MAT range of about -1 to -5 deg C) has been reported previously in Manitoba (see Camill 2005 Climatic Change).

Our response: This reference is now included.

Lines 231-234: It's not clear from Fig 1 or 5A how these sites relate to climate. The authors state in the Fig. 5A caption that the sites are ordered from left to right according to decreasing MAT, but mean air temps are not reported in Table S1 for two of the four sites (Trout Lake or Sixtieth). The MATs reported in Table S1 (burned MAT/unburned MAT) are as follows:

Sixtieth = NA/NA
FT Simp = -0.15/-0.81
Trout = NA/NA
Zama = NA, -1.57

Is it possible to fill these gaps to better support the climate-thermokarst relationship?

Our response: The data in Table S1 indicates the mean annual temperature at the field sites, not the remote sensing sites. The Zama site is the only site where we had field and remote sensing analysis done for the very same location. The fort Simpson field site and remote sensing sites were ~25 km apart. No field site was close to the Trout Lake or 60th remote sensing sites.

We thus do not have direct measured air temperatures from all the four remote sensing sites. However, the Zama site with the lowest coverage of young thermokarst bogs was also our coldest field site (-3.1°), and it was also the site located at the highest elevation (~300 m higher than the lower sites), and is located within a low subarctic ecoregion. Trout Lake site is located at similar elevation as Zama, and is thus likely to have a cooler climate than the lower elevation FtSimpson and 60th parallel sites. The field site at FtSimpson accordingly had a mean annual temperature of -0.8°C. Given the lack of direct data to support our statement of differences in climate, we have now cautioned our interpretation of the differences between remote sensing sites, but still speculate that likely differences between sites is driven by differences in climate, and this is indicated in the figure legend L702-703:

“Sites are ordered left to right by likely decreasing mean annual air temperature, see text for justification.”

And this justification is found in the results section, L201-211:

“There was, however, a large variability in young thermokarst bog coverage among unburned sites, between 2.7 and 10.5%, with the lowest coverage at the higher elevation sites, Zama and Trout Lake (Supplementary Table 2). The Zama and Trout Lake sites were located at elevations ~300 m higher than the two other sites, and are indicated to have low subarctic climate in contrast to the lower elevation sites that are located in high boreal or mid-boreal ecoregions. Fied data also confirmed that the Zama site had a colder climate than the Fort Simpson site during the year of our study, at -0.8 and -3.1˚C.

While no direct climate data for the 60th parallel site was available, this site was located both at a low elevation and in the southernmost part of the study region, and would thus be expected to have the warmest climate, explaining the greatest young thermokarst bog coverage within the unburned areas among our four sites."

Another point: If the temperature range is only from 0 to -1.57 deg C, the authors need to consider that this is a relatively narrow temperature range on the warm end of the climate/permafrost spectrum for interpreting climate-thermokarst dynamics. If the authors were to push northwards into regions where MAT was below -2, -5, -7 deg C, for instance, they may observe less thermokarst even with burning. This is where a climate x fire interaction would likely show up. While the results in this study are interesting and useful, broader generalizations between climate, fire, and thermokarst may not be warranted--or done so with caution since this study only focuses on one narrow climatic range for the warm end of the discontinuous permafrost zone.

Our response: Our climate range is a bit broader than indicated in comment, ranging from 0 to -3.1°C (Supplementary Table 1) in the year of study at our field sites, which likely also is the same climate range as for our remote sensing sites, see discussion above.

Overall, we do believe that our sites are representative of the chosen study region. Peat plateaus have little variation in terms of vegetation communities, structure, and ground microtopography within the study region. Thick peat accumulation and permafrost conditions are strong controls on site hydrology and nutrient availability, and thus ensures the similarity between peat plateau sites. In short, peat plateaus are probably one of the most suitable ecosystems for conducting space-for-time chronosequence studies. Furthermore, while all our study locations were found in the southern part of the identified study region, some of these locations (e.g. the Zama and Trout lake locations) were located at higher elevation (~300 to 400 m higher than elevations in the northern part of the study region). Elevation is a strong control on the local climate and thus each of the three Level III ecoregions found within the study region (mid-boreal, high boreal, and low subarctic) were represented by at least one study location. We have included a sentence in the methods to emphasize this (L333-337):

"While all locations were found in the southern part of the identified study region, each of the three level III ecoregions of within the study region (mid-boreal, high boreal, and low subarctic ecoregions) were represented by at least one study location, with the colder low subarctic climate ecoregion represented in southern part of the study region by a location at higher elevation (Zama)."

We have, however, made several changes to emphasize that the magnitude and duration of effects of wildfire on peatland permafrost stability found in this study should not be directly extrapolated to other regions, given differences in climate, peatland development history, and fire regimes:

L12-14: "In this study of western Canadian permafrost peatlands, we assess impacts of wildfire on soil thermal regime and rate of thermokarst bog expansion resulting from complete permafrost thaw."

L265-268: "In this study, we showed that wildfire in boreal peatlands within the discontinuous permafrost zone cause permafrost thaw through active layer deepening

and talik expansion on peat plateaus, but also through accelerated thermokarst bog development along peat plateau edges."

L301-304: "While the magnitude of effects from wildfire are likely to differ depending on the regional climate, peat plateaus under similar climates and fire regimes as described in this study are widespread also in boreal Alaska and in the Hudson Bay lowlands."

Fig 5. The panels need to be labeled A and B.

Our response: This has been incorporated in the revisions.

Lines 236-238: This statement may help, but I'm not sure as to what landscapes we're talking about--is it the four peatland sites or all of them?

Our response: This is referring to the four peatlands that were used in the remote sensing analysis. The manuscript has been updated to reflect this, Line 213-216:

"Despite the apparent influence of climate on young thermokarst bog coverage, we found a consistent effect of wildfire among the four peatlands where landscape classification was done, with 3.4 ±0.5% (±1 SD) greater young thermokarst coverage in burned than unburned peatland parts."

Lines 256-272: This is an interesting analysis that, in part, addresses some of the concerns raised previously, and the assumptions made are necessary (although see next point below), but as mentioned earlier, why not let the remote sensing data across all burn age classes inform this analysis?

Our response: The rationale for our choice of remote sensing sites has been discussed above. As mentioned, we believe our choice of sites allowed us to characterize most of the cumulative effect of wildfire on thermokarst bog development, but that it may be a slightly conservative estimate as we don't know if the effect of fire lasts >30 years.

Lines 263-264: The rate of transition of young to mature thermokarst bogs is dependent on the local hydrology, peat accumulation rates, and bulk density increases in the thermokarst bogs. These can lead to variable transitions and ought to be mentioned as caveats in the methods.

Our response: This is accounted for, and it is explicitly shown in Fig 5b (the uncertainty is shaded and is largely driven by the uncertainty of the period of persistence for young thermokarst bogs). Furthermore, Supplementary Table 5 reports the variability in how long the young thermokarst bog stage persists, as found at a number of sites in the study region. We further highlight this assumption and the data that supports it in the methods, L442-446:

"Secondly, we assumed that young thermokarst bogs persist in the landscape 100 ± 50 years (± 95% CI) before developing into mature thermokarst bogs. This assumption is based on 14C and Pb dating of peat cores (Table S5), and implies that all young thermokarst bogs currently present in peatland developed <150 years ago."

Lines 292, 297-299: Be cautions of overinterpretation. "Clearly" in line 292 is an overstatement. Without the analysis of thaw % by fire age across all landscape fire age classes, the authors

need to be more cautious with these interpretations. Line 297 needs to be restated. Peat plateaus can burn but not thaw, especially in colder regions.

Our response: Changes have been made. The work 'clearly" has been removed, and the first sentence of the discussion has been revised to qualify our findings to the study region (L265-268):

"In this study, we showed that wildfire in boreal peatlands within the discontinuous permafrost zone cause permafrost thaw through active layer deepening and talik expansion on peat plateaus, but also through accelerated thermokarst bog development along peat plateau edges."

We also caution the extrapolation of our findings to colder regions, L301-304:

"While the magnitude of effects from wildfire are likely to differ depending on the regional climate, peat plateaus under similar climates and fire regimes as described in this study are widespread also in boreal Alaska and in the Hudson Bay lowlands."

Lines 317-323: The citations for CH_4, CO_2, and peat accumulation changes with thermokarst could be improved. There is a large body of research on each of these.

Our response: We have added 2 studies that review aspects of carbon cycling in response to permafrost thaw.

Lines 326-334: As alluded to above, the authors need to be cautions when generalizing beyond the southern edge of the sporadic discontinuous permafrost zone. Permafrost in colder regions will likely not respond, at least currently/initially, as that in this study. While it is tempting to extrapolate these results to other boreal areas and the tundra, that would almost certainly overestimate the effect of fire on thaw. Although beyond the scope of this manuscript, what is needed, instead, is a probabilistic framework that describes the likelihood of thaw given a burn across a broad MAT gradient (0 to -8 deg C). Until this kind of analysis is performed, the peatland scientific community needs to be cautions in extrapolating dynamics from the southern boundary across the pan-boreal/arctic regions.

Our response: As described above, we do believe that our results are representative for the study region, which is defined by the discontinuous permafrost zone in western Canada. We agree that extrapolation of our results to colder climate should be done with caution, and we have made several changes to emphasize that the magnitude and duration of effects of wildfire on peatland permafrost stability found in this study should not be directly extrapolated to other regions, given differences in climate, peatland development history, and fire regimes:

L12-14: "In this study of western Canadian permafrost peatlands, we assess impacts of wildfire on soil thermal regime and rate of thermokarst bog expansion resulting from complete permafrost thaw."

L265-268: "In this study, we showed that wildfire in boreal peatlands within the discontinuous permafrost zone cause permafrost thaw through active layer deepening and talik expansion on peat plateaus, but also through accelerated thermokarst bog development along peat plateau edges."

L301-304: "While the magnitude of effects from wildfire are likely to differ depending on the regional climate, peat plateaus under similar climates and fire regimes as described in this study are widespread also in boreal Alaska and in the Hudson Bay lowlands."

Line 383: 3-m

Our response: This has been incorporated in the revisions.

Line 425: What is the resolution of the remotely sensed data? It needs to be fairly high to be able to resolve decadal-scale changes in thermokarst.

Our response: The satellite image spatial resolution (WorldView2) is 0.6 m, which is stated in the methods section (L407), and in figure 4 legend (L693). This is sufficient to estimate areas of young thermokarst bogs, since these features often are ~2 to 10 m wide along peat plateau edges. Our field validation indicated that there was no bias in the satellite image classification and thus we do not believe that there is a low or high bias in our estimates of young thermokarst bog coverages.

Reviewers' comments:

Reviewer #1 (Remarks to the Author):

In the revised version of the manuscript the authors have adequately addressed all minor questions and suggestions I had. I recommend that this paper be published.

Reviewer #3 (Remarks to the Author):

I have reviewed the revised manuscript, and the authors did a good job incorporating reviewer feedback. This version is clearer and better supported. I raise two points below: one relatively minor correction and a second, more substantive concern with one of the statistical methods that is important to address.

Lines 64-66:

Please change this: "Previous studies have used repeat aerial photography and satellite image analysis to show that the rate of thermokarst bog development has increased over the last few decades due to warming."

To this: "Previous studies have used repeat aerial photography, satellite image analysis, and tree ring analysis to show that the rate of thermokarst bog development has increased over the last few decades due to warming."

Lines 216-219: A few thoughts about the ANOVA used here:

(1) Statistical assumptions

Note: The comments below assume a fairly simple 2-way ANOVA model was used in this case. I could not tell (or I missed it) from the methods section. My apologies to the authors if this is a misinterpretation.

Since there are four sites, each with 30-65, 250 x 250-m plots in burned and 30-65, 250 x 250-m plots in unburned parts, the unit of analysis for the ANOVA appears to be the level of plots (the "n" in Supplementary table 2). Site and fire are being used as treatment effects. If this interpretation of the sampling design is correct, a fairly substantive statistical challenge arises:

The 30-65 plots are actually pseudoreplicates within site--i.e., we don't have 30-65 samples drawn from independent sites. If we are treating site as an approximate temperature proxy and attempting to gain information about the importance of site as a factor, this technically violates the IID assumptions of parametric ANOVA models, most likely inflating the F values (and deflating the p values) reported in Supplementary table 6. Simply put, the sample size (n) is artificially too high whenever pseudoreplication is present.

If site is intended to be a meaningful factor, there is no easy way around this challenge given that the authors were limited logistically to the four study sites. This challenge is substantial enough that it would be preferred if the authors could explore the alternate statistical approaches below for this case (A and B are likely more robust than C):

(A) Technically, site should be abandoned as a stand-alone factor since it is not adequately replicated. In this case, site becomes the unit of analysis/replicate (n = 4) and the 30-65 pseudoreplicates are averaged to generate a mean value per site for burned and unburned areas.

Then the effects of burned/unburned could then be handled with an appropriate t test. This approach may also be warranted because the lack of mean annual air temps for some of the remote sensing sites makes it difficult to ascribe specific temperature variation to the site variable.

(B) If site is retained as an independent variable, perhaps a more appropriate ANOVA model could be used. One option might be a nested mixed-effects ANOVA, with site as a random effect, plots nested within site, and fire as the only fixed effect. Since there is only one fixed effect in this model, there would be no exploration of interactions.

(C) If site is retained as an independent variable, perhaps a nonparametric resampling method could also be explored.

The alternate approaches are not intended to add a burden of significant new data analysis but are meant to help the authors verify the conclusions based on the results of the original 2-way ANOVA. The interpretation of site as a unique factor is problematic here due to the pseudoreplication challenge and lack of available mean annual air temperaure for some of the sites. I'm pretty certain that, using an alternative modeling approach, the p values would show a lower level of significance than what is currently reported.

(2) Potential interactions

Note: This comment may be irrelevant depending on the outcome of suggestion (1) above.

The authors state, "Accordingly, a two-way ANOVA of young thermokarst bog coverage found strong effects of both site and fire history but no significant interactive effect (Supplementary Table 6)."

but then follow up with

"As such, the relative effect of wildfire on rate of thermokarst bog expansion appeared much greater at colder sites (Fig. 5a)."

Maybe I'm not reading this correctly, but these two statements appear to be contradictory. If the ANOVA says that there is no interaction between site and fire, this would caution against saying that the difference between burn and unburned areas is different across sites. Put another way, this means that the burned/unburned difference in the coldest site is not statistically different than the burned/unburned difference in the warmest site. The phrase "much greater" becomes statistically irrelevant when there is no significant interaction.

Response to a reviewer comments on "Wildfire as a major driver of recent permafrost thaw in boreal peatlands." by Gibson et al.

We would like to thank the reviewers for reviewing our changes following the first review round, and for providing additional comments. We have now addressed the few remaining comments, which did not lead to any substantial changes to our overall interpretation of the data. Below we address specific revisions in response to each reviewer comment. Line number references refer to those in the revised manuscript.

Reviewers' comments:

Reviewer #1 (Remarks to the Author):

In the revised version of the manuscript the authors have adequately addressed all minor questions and suggestions I had. I recommend that this paper be published.

Reviewer #3 (Remarks to the Author):

I have reviewed the revised manuscript, and the authors did a good job incorporating reviewer feedback. This version is clearer and better supported. I raise two points below: one relatively minor correction and a second, more substantive concern with one of the statistical methods that is important to address.

Lines 64-66:

Please change this: "Previous studies have used repeat aerial photography and satellite image analysis to show that the rate of thermokarst bog development has increased over the last few decades due to warming."

To this: "Previous studies have used repeat aerial photography, satellite image analysis, and tree ring analysis to show that the rate of thermokarst bog development has increased over the last few decades due to warming."

Our response: That's correct, and this change is reflected in the updated manuscript (L64-66)

Lines 216-219: A few thoughts about the ANOVA used here:

(1) Statistical assumptions

Note: The comments below assume a fairly simple 2-way ANOVA model was used in this case. I could not tell (or I missed it) from the methods section. My apologies to the authors if this is a misinterpretation.

Since there are four sites, each with 30-65, 250 x 250-m plots in burned and 30-65, 250 x 250-m plots in unburned parts, the unit of analysis for the ANOVA appears to be the level of plots (the "n" in Supplementary table 2). Site and fire are being used as treatment effects. If this interpretation of the sampling design is correct, a fairly substantive statistical challenge arises:

The 30-65 plots are actually pseudoreplicates within site--i.e., we don't have 30-65 samples drawn from independent sites. If we are treating site as an approximate temperature proxy and attempting to gain information about the importance of site as a factor, this technically violates the IID assumptions of parametric ANOVA models, most likely inflating the F values (and deflating the p values) reported in Supplementary table 6. Simply put, the sample size (n) is artificially too high whenever pseudoreplication is present.

If site is intended to be a meaningful factor, there is no easy way around this challenge given that the authors were limited logistically to the four study sites. This challenge is substantial enough that it would be preferred if the authors could explore the alternate statistical approaches below for this case (A and B are likely more robust than C):

(A) Technically, site should be abandoned as a stand-alone factor since it is not adequately replicated. In this case, site becomes the unit of analysis/replicate (n = 4) and the 30-65 pseudoreplicates are averaged to generate a mean value per site for burned and unburned areas. Then the effects of burned/unburned could then be handled with an appropriate t test. This approach may also be warranted because the lack of mean annual air temps for some of the remote sensing sites makes it difficult to ascribe specific temperature variation to the site variable.

(B) If site is retained as an independent variable, perhaps a more appropriate ANOVA model could be used. One option might be a nested mixed-effects ANOVA, with site as a random effect, plots nested within site, and fire as the only fixed effect. Since there is only one fixed effect in this model, there would be no exploration of interactions.

(C) If site is retained as an independent variable, perhaps a nonparametric resampling method could also be explored.

The alternate approaches are not intended to add a burden of significant new data analysis but are meant to help the authors verify the conclusions based on the results of the original 2-way ANOVA. The interpretation of site as a unique factor is problematic here due to the pseudoreplication challenge and lack of available mean annual air temperature for some of the sites. I'm pretty certain that, using an alternative modeling approach, the p values would show a lower level of significance than what is currently reported.

Our response: Issues with pseudoreplication is a recurring issue when investigating effects of large scale disturbances like wildfire, since it almost inevitably prevents proper replication. A recent review/comment on the issue was recently published by Davies and Gray: "Don't let spurious accusations of pseudoreplication limit our ability to learn from natural experiments (and other messy kinds of ecological monitoring)", Ecology and Evolution, 2015, 5(22): 5295–5304. We were fortunate in this study to be able to find 4 partially burned peatlands in the study region, which burned at a similar time and that were large enough that we could carry out the spatial analysis as described. We thus have been aware of the potential pseudoreplication of our approach, and in particular we tried to convey that the differences found between sites using the 2-way ANOVA could not directly be attributed to differences in local climate – that the cause of differences between paired sites was only an inference.

In order to avoid any issue of pseudoreplication, however, we have made changes that follow suggestion (A) above – i.e. simplifying the statistics to a pairwise t-test using the average young thermokarst bog coverage of burned and unburned parts within each site. Despite the low replication (n =4), we arrive at a highly significant effect of wildfire (p = 0.007). We have also made changes to only discuss differences between sites qualitatively – where we make the observations that unburned sites at higher elevation had lower young thermokarst bog coverages than unburned sites at lower elevation. The revised paragraph for these results now read:

L200-222: "Average coverage of young thermokarst bogs within burned and unburned peatland parts was 8.6% and 5.3%, respectively, and the average difference between burned and unburned parts of paired sites was found to be 3.4 ±0.5% (±1 SD) (Fig. 5a). A pairwise t-test indicated a significant influence of fire on young thermokarst bog coverage (t = -10.889, p < 0.01) when comparing average young thermokarst bog coverage in burned and unburned parts of the four paired sites. While the effect of wildfire was largely consistent between sites, we did observed a large variability in average young thermokarst bog coverage between unburned sites, ranging from 2.7 to 10.5% (Fig. 5a). We note that the lowest young thermokarst bog coverage was found at the higher elevation sites, Zama and Trout Lake (Supplementary Table 2). These sites are located at elevations ~300 m higher than the two other sites, and are indicated to have low subarctic climate in contrast to the lower elevation sites that are located in high boreal or mid-boreal ecoregions18. Field data confirmed that the Zama site had a colder climate than the Fort Simpson site during the year of our study, at -0.8 and -3.1˚C. While we have no direct climate data for the 60th Parallel site was available, this site was located both at a low elevation and in the southernmost part of the study region, and would thus be expected to have the warmest climate, thus possibly explaining the greatest young thermokarst bog coverage within the unburned areas among our four sites. This implied effect of climate on thermokarst bog development in unburned peatland parts23 contrasted with the lack of an observed difference in soil thermal regimes among unburned sites (Fig. 3). The greater young thermokarst bog coverage in burned than unburned parts at the Zama and Trout sites (+100-150% greater coverage in burned than unburned parts) than at the 60th Parallel and Fort Simpson sites (+30-70% greater coverage in burned than unburned parts) thus suggests that wildfire has had a relatively more pronounced influence on thermokarst bog expansion at colder sites (Fig 5a). "

We also made appropriate changes to reflect this in the methods section (L438-442), in the figure legend for figure 5 (L708-709), and removed a table from the supplementary information.

(2) Potential interactions

Note: This comment may be irrelevant depending on the outcome of suggestion (1) above.

The authors state, "Accordingly, a two-way ANOVA of young thermokarst bog coverage found strong effects of both site and fire history but no significant interactive effect (Supplementary Table 6)."

but then follow up with

"As such, the relative effect of wildfire on rate of thermokarst bog expansion appeared much greater at colder sites (Fig. 5a)."

Maybe I'm not reading this correctly, but these two statements appear to be contradictory. If the ANOVA says that there is no interaction between site and fire, this would caution against saying that the difference between burn and unburned areas is different across sites. Put another way, this means that the burned/unburned difference in the coldest site is not statistically different than the burned/unburned difference in the warmest site. The phrase "much greater" becomes statistically irrelevant when there is no significant interaction.

Our answer: We have clarified this section. What we mean is that the effect of fire (which increased young thermokarst bog coverage by ~3% in all sites) represents a greater effect at the higher elevation sites which only had ~2.5% young thermokarst bog coverage in the unburned sites, than at the lower elevation sites where the unburned sites had 10% young thermokarst bog coverage. The revised statement reads:

“The greater young thermokarst bog coverage in burned than unburned parts at the Zama and Trout sites (+100-150% greater coverage in burned than unburned parts) than at the 60th Parallel and Fort Simpson sites (+30-70% greater coverage in burned than unburned parts) thus suggests that wildfire has had a relatively more pronounced influence on thermokarst bog expansion at colder sites (Fig 5a). “
